# Regulation of the cardiac sodium pump

**DOI:** 10.1007/s00018-012-1134-y

**Published:** 2012-09-07

**Authors:** W. Fuller, L. B. Tulloch, M. J. Shattock, S. C. Calaghan, J. Howie, K. J. Wypijewski

**Affiliations:** 1 Division of Cardiovascular and Diabetes Medicine, Medical Research Institute, College of Medicine Dentistry and Nursing, University of Dundee, Dundee, UK; 2 Cardiovascular Division, King’s College London, London, UK; 3 School of Biomedical Sciences, University of Leeds, Leeds, LS2 9JT UK; 4 Division of Cardiovascular and Diabetes Medicine, Ninewells Hospital, Mail Box 12, Level 5, Dundee, DD1 9SY UK

**Keywords:** Sodium pump, Ion transport, Phospholemman, FXYD, Heart, Intracellular sodium, Protein kinase A, Protein kinase C, Palmitoylation

## Abstract

In cardiac muscle, the sarcolemmal sodium/potassium ATPase is the principal quantitative means of active transport at the myocyte cell surface, and its activity is essential for maintaining the trans-sarcolemmal sodium gradient that drives ion exchange and transport processes that are critical for cardiac function. The 72-residue phosphoprotein phospholemman regulates the sodium pump in the heart: unphosphorylated phospholemman inhibits the pump, and phospholemman phosphorylation increases pump activity. Phospholemman is subject to a remarkable plethora of post-translational modifications for such a small protein: the combination of three phosphorylation sites, two palmitoylation sites, and one glutathionylation site means that phospholemman integrates multiple signaling events to control the cardiac sodium pump. Since misregulation of cytosolic sodium contributes to contractile and metabolic dysfunction during cardiac failure, a complete understanding of the mechanisms that control the cardiac sodium pump is vital. This review explores our current understanding of these mechanisms.

**Electronic supplementary material:**

The online version of this article (doi:10.1007/s00018-012-1134-y) contains supplementary material, which is available to authorized users.

## Introduction

In 1997, a share of the Nobel Prize in Chemistry was awarded to Jens Christian Skou for his 1957 discovery of the Na, K ATPase [1]. This ubiquitous P-type ATPase links the hydrolysis of ATP to the cellular export of three sodium ions and import of two potassium ions against their electrochemical gradients. Subsequent work by Skou and countless other research groups worldwide established the Na/K ATPase (Na pump) as an indispensable means of active membrane transport in essentially every eukaryotic single and multi-cellular organism, and the molecular target of the foxglove extracts digitalis and digoxin, in clinical use for heart failure since the 18th century.

While the use of Na pump-inhibiting cardiotonic steroids for the management of heart failure is now restricted to only a small subset of patients [2], our understanding of the biology and regulation of the Na pump has blossomed. In the past 5 years, the discovery [3] and refinement [4–7] of crystal structures in potassium and ouabain-bound states has further advanced our understanding of the structure–function relationship of this much-studied macromolecular complex. The Na pump is subject to multiple regulatory mechanisms in essentially every tissue in which it is expressed that are beyond the scope of this review, which will focus solely on the regulation of the cardiac enzyme.

### Quaternary structure

The Na pump is a multi-subunit enzyme with a minimum requirement for an α and β subunit to form a functional pump. The ~100-kDa α subunit is the catalytic core of the enzyme, containing the binding sites for sodium, potassium, and ATP as well as cardiotonic steroids such as ouabain. It requires an obligatory association with a β subunit to traffic through the secretory pathway to the plasma membrane [8, 9]. The discovery of a third subunit, the γ subunit in the kidney [10], eventually led to the realization that a third protein may more generally form part of the pump complex [11]. Whether this third member of the complex, named a FXYD protein for the conserved extracellular phenylalanine-X-tyrosine-aspartate motif, is a constant or occasional companion of the pump has not been rigorously investigated to date. The existence of four isoforms of the α subunit, three isoforms of β, and seven FXYD proteins (as well as splice variants of the γ subunit [12]) in mammalian genomes can theoretically support the assembly of over 100 functionally different Na pumps to fulfill different physiological requirements.

#### Cardiac subunit composition

Although four isoforms of the α subunit have been identified, only α1 and α2 are reportedly expressed at significant levels in cardiac myocytes [13, 14]. That said, we [15] and others [16] readily detect α3 subunit expression in cardiac tissue (which may not reflect a myocyte-derived pool), and the α3 subunit is reported to replace the α2 subunit in experimental models of heart failure [17]. Both the α1 [18] and α2 [14] subunits of the Na pump are functionally linked to the Na/Ca exchanger (NCX) in ventricular myocytes, however the subcellular distribution of these two isoforms is different, with the α2 subunit found more concentrated in t-tubular membranes than the α1 subunit [19]. This has led to some proposing different physiological roles for these two subunits [14, 20], although this hypothesis has been challenged [21]. Experiments in transgenic animals in which the ouabain affinities of α1 and α2 isoforms of the pump are reversed clearly indicate that both α1 and α2 are functionally and physically coupled to NCX in the heart [18]. Recent experiments using the same transgenic model suggest that the functional coupling of α2-containing pumps to NCX has a greater impact on myocyte calcium handling than the functional coupling of α1-containing pumps [22]. When α1- or α2-containing pumps are selectively blocked to give similar rises in intracellular sodium, only α2 block increases calcium transient amplitude. This suggests that α2 pumps control sodium and therefore calcium in sarcolemma/sarcoplasmic reticulum microdomains via NCX, and α1 pumps are responsible for maintaining a separate (possibly global) pool of sodium [22].

In the mouse, approximately 70 % of functional α2 and 40 % of α1 subunits are t-tubular, even though the t-tubule membranes represent only 30 % of total surface area [19]. The contribution of the α1 isoform dominates the α2 in all surface membrane compartments, but it is much less dominant in the t-tubules [19]. A similar functional concentration in t-tubules [23] and isoform distribution is reported for the rat [24]. Prevailing opinion therefore currently favors the concept that while both α1 and α2 subunits of the pump are involved in regulation of excitation–contraction (E–C) coupling, α2 containing pumps are principally concerned with regulation of contractility, and α1 containing pumps control both contractility and bulk intracellular sodium. There are undoubted differences in the manner in which α1 and α2 pumps are regulated hormonally (see “Basal phosphorylation of phospholemman”), and the biochemical basis of the differential targeting of Na pump α1 and α2 containing enzymes has not yet been established.

The principle β subunit found in cardiac muscle is β1 (although we also routinely detect the β3 subunit in proteomic screens from ventricular myocytes [15]), and the principal FXYD protein is phospholemman (PLM). FXYD5 (RIC) has also been reported to be present in homogenates from whole hearts [25], but whether this derives from a myocyte or non-myocyte population remains to be investigated.

### Importance of pump regulation in the heart

In excitable tissues, the activity of the plasmalemmal Na pump is vital for the maintenance of normal electrical activity and ion gradients. In cardiac muscle, the transarcolemmal sodium gradient established by Na pump activity is essential not only for generating the rapid upstroke of the action potential but also for driving a number of ion exchange and transport processes critical for normal cellular function, ion homeostasis, and the control of cell volume. These sodium-dependent membrane transporters include those responsible for the regulation of other ions (such as NCX, Na/H exchanger, and Na-HCO3 cotransporter [26]), as well as those involved in the movement of substrates and amino acids (see Table 1) [27]. For example, by controlling steady-state intracellular sodium, the pump determines the set-point for intracellular calcium (Ca) via NCX, which in turn determines the sarcoplasmic reticulum (SR) calcium content. Precise control of cytosolic and SR calcium concentrations is essential in maintaining cardiac output: derangement of calcium handling is a primary cellular cause of contraction abnormalities and heart failure. Interventions that influence either the set point of the Na pump, or indirectly the transarcolemmal sodium gradient, can therefore profoundly affect myocardial function. In essence, the Na pump indirectly controls myocardial contractility [28].

**Table 1:** Mammalian cell surface translocators whose activity relies on the transmembrane Na gradient

Translocator	Stoichiometry	Charge flux
Ions		
Na channel	N/A	−ve
Na/Ca exchanger	3:1	−1
Na/H exchanger	1:1	Neutral
Na/Mg exchanger	2:1	Neutral
Na/K/Cl co-transporter	1 + 1 + 2	Neutral
Na/HCO_3_ co-transporter	1 + 2	+1
Na/I co-transporter	2 + 1	−1
Substrates		
Na/glucose co-transporter	2 + 1	−2
Na/mannose co-transporter	1 + 1	−1
Na/Cl/creatine co-transporter	2 + 1 + 1	−1
Na/succinate co-transporter	3 + 1	−1
Amino acids		
Na/Cl/taurine co-transporter	3 + 1 + 1	−2
Na/glutamate/K exchanger	3 + 1:1	−1
Na/Cl/glycine co-transporter	3 or 2 + 1 + 1	−2 or −1
Na/alanine co-transporter	1 + 1	−1
Na/Cl/GABA co-transporter	2 + 1 + 1	−1
Na/Cl/proline co-transporter	3 + 1 + 1	−1
Na/arginine co-transporter	1 + 1	−2
Na/glutamine/H exchanger	2 + 1:1	−1
Others		
Na/ascorbate co-transporter	2 + 1	−1
Na/citrate co-transporter	3 + 1	−1
Na/monocarboxylate co-transporter	2 + 1	−2
Na/Pi co-transporter	3 + 1	−1
Na/sulphate co-transporter	3 + 1	−1
Na/bile acid co-transporter	2 + 1	−1
Na/nucleoside co-transporters	1 + 1	Neutral
Na/inositol co-transporter	1 + 1	−1
Na/Cl/dopamine co-transporter	2 + 1 + 1	−2
Na/Cl/noradrenaline co-transporter	1 + 1 + 1	−1
Na/Cl/serotonin/K exchanger	2 + 1 + 1:1	−1
Na/carnitine co-transporter	1 + 1	−1

By convention, inward positive charge movement is classified as a negative flux. Modified from [29]

## Pump regulation by the prevailing cellular environment

### ATP

The *K*_m_ of the cardiac Na pump for intracellular ATP has variously been estimated to range from 0.46 mM (embryonic chick hearts [30]) to 0.21 mM (dog sarcolemmal vesicles [31]) to 94 μM (giant patches from guinea-pig, rabbit, and mouse myocytes [32]). In rat ventricular myocytes, intracellular ATP is generally high enough to be non-limiting for the Na pump: even following metabolic inhibition, ATP concentration only declines to a limiting concentration at about the time of onset of rigor-contracture [33]. Interestingly, there is evidence that the pump is functionally coupled to glycolytic rather than oxidatively generated ATP [34], much as has been reported for other sarcolemmal ion transporters [35], although the molecular basis for this remains to be established.

### Intracellular sodium

The affinity of the cardiac pump for sodium has been reported for a number of species and using a variety of techniques. The *K*_m_ for sodium has variously been estimated as: 9 mM (dog sarcolemmal vesicles [31]), 14 mM (sheep Purkinje fibers [36]), 11 mM (guinea-pig ventricular myocytes [37]), 19 mM (rabbit ventricular myocytes [38]), 19 mM (mouse ventricular myocytes [39]). Inter-species variations in resting intracellular sodium concentrations in ventricular myocytes have also been reported [40], but what is beyond doubt is that intracellular sodium sits close to the sodium affinity of the pump, such that small changes in intracellular sodium elicit large changes in pump activity. That said, the sodium concentration seen by the pump may actually differ from bulk resting cytosolic sodium.

#### Sub-sarcolemmal barriers and fuzzy spaces

The idea that sub-sarcolemmal sodium gradients not only exist but may dynamically change to influence E–C coupling was first proposed by Lederer et al. [41] and Leblanc and Hume [42] in 1990. This raised the possibility that the Na pump may both respond to, and influence, limited pools of sodium in a sub-sarcolemmal space. There is now ample evidence for a diffusional barrier between a sub-sarcolemmal pool of sodium available to the pump and the bulk cytosolic pool in cardiac muscle [43–48]. Hence, while sodium availability is one of the principal determinants of pump activity in all cell types, in cardiac muscle in particular, the activity of co-localized sodium influx pathways which “charge” this sub-sarcolemmal pool may be an important controller of the pump. Chief among these is NCX, which has been suggested to be functionally coupled to the pump [14, 18], and whose expression and activity is significantly elevated in some experimental models of heart failure (for example [49, 50]). Hence, while the pump acutely controls NCX (see above), changes in NCX-driven sodium transport may also drive long-term changes in flux through the pump when, for example, downregulation of SERCA in the failing heart causes myocytes to become more dependent on NCX to remove calcium during diastole [49, 50].

While the pump may functionally interact with NCX, the situation may be more complex. As discussed above (“Cardiac subunit composition”), α1 and α2-containing isoforms may be both spatially and functionally separate. α2 expression is concentrated in the t-tubules, along with other key components of E–C coupling, while α1 is more evenly distributed across the sarcolemma and has been suggested to play a more “housekeeping” role in regulating bulk cytoplasmic sodium [14, 18–20, 22]. Thus, it may be inappropriate to think of the cell as two simple compartments, bulk cytosol and sub-sarcolemmal space, but rather as multiple compartments where co-localization of ion transporters creates microdomains of locally controlled sodium. However, in this regard, both Weber et al. [51] and Silverman et al. [48] failed to detect evidence that the cardiac sodium current elevates sodium in a sub-sarcolemmal space sensed by either NCX or NKA at physiological potentials. While this leaves open the possibility that NCX and NKA occupy the same sub-sarcolemmal compartment, it seems that sodium in this compartment does not change in the milliseconds following sodium entry via the fast inward current. While this may be the case in healthy single cardiac myocytes under controlled experimental conditions, there is ample evidence that other sodium influx pathways are also upregulated in the failing myocardium [52, 53], which leads to elevated steady-state bulk sodium in failing myocytes, and therefore higher steady-state flux through the pump [52]. Added to this, changes in the expression of pump catalytic and regulatory subunits contribute to altered pump function in heart failure [16].

### Membrane potential

The activity of the Na pump is strongly influenced by membrane voltage [24, 37, 38, 54]. Thus, during the action potential, even if the trans-sarcolemmal ion gradients remain unchanged, pump activity both responds to, and can influence, membrane potential. The voltage-dependence of the pump typically shows some inward rectification at positive potentials [37, 38, 54]. At rest, pump inhibition depolarizes the membrane by only a few millivolts as the relatively small pump current is dominated by the large potassium conductance of the resting membrane (*I*_K1_). However, during the plateau of the action potential, the input impedance of the membrane is significantly raised (due to the inward rectification of *I*_K1_) and hence the pump current, which is itself increased by depolarization, may have a more significant impact on membrane potential with pump stimulation shortening, and inhibition prolonging, the duration of the action potential [55].

The influence of voltage on pump current differs between the two main cardiac isoforms [24, 56]. While both subunits are activated by voltage, and show inwardly rectifying current–voltage relationships, the voltage-dependence of the α2 subunit is more strongly influenced by extracellular potassium and sodium [56, 57]. Since the α2 isoform is localized to t-tubules, where extracellular diffusion may be limited, it is possible that at high heart rates where potassium may accumulate in extracellular clefts, this may preferentially affect the activity of the α2 isoform. The steeper voltage-dependence (associated with accumulation of potassium in the t-tubules) may therefore preferentially enhance the activity of the α2 isoform during the plateau of the action potential. The contribution of this isoform to overall current is small (even in the t-tubule), but it is possible that this preferential activation may affect local sub-sarcolemmal sodium in the t-tubule microdomain and have an impact on E–C coupling.

## Pump regulation by intracellular signaling pathways

### Adrenergic signaling pathways

The functional link between the adrenergic system and the cardiac pump is well established, but remarkably, after many decades of research, there is still disagreement about the functional consequences of adrenoceptor activation on pump activity. In general activation of PKA (via β1-adrenoceptors linked to adenylate cyclase) and PKC (via α1 adrenoceptors linked to phospholipase C) increases stroke volume in ventricular muscle via phosphorylation of L-type calcium channels, the ryanodine receptor and phospholamban [58]. Pump inhibition is classically positively inotropic as it reduces calcium extrusion by NCX and therefore increases calcium uptake to the SR. Activation of both adrenoceptor pathways is likely, however, to also involve a concomitant increase in heart rate and hence sodium influx. Thus, while Na pump activation under such circumstances might be expected to limit the positive inotropy (by limiting the rate-induced rise in sodium) this effect may be minimal (in the face of the profound increase in the calcium transient) and is probably a price worth paying for maintaining calcium extrusion via NCX and hence improving relaxation and diastolic function. Indeed, pump activation by the adrenergic system appears to be essential to protect against sodium and calcium-overload-induced arrhythmias (discussed in “The functional role of phospholemman phosphorylation”).

#### Protein kinase A

Most laboratories report activation of the pump by PKA agonists in the heart [39, 59–68], some report inhibition [69, 70], some no effect [71], while others report both activation and inhibition depending on experimental design [72]. The details of the molecular events underlying pump regulation by PKA will be discussed below. The disagreement in the functional effect of PKA on pump activity is difficult to reconcile between different laboratories and different techniques, but is perhaps best explained by the relatively overlooked work of Gao et al. [72], who investigated pump activity using the whole cell patch clamp technique in guinea-pig ventricular myocytes. They found that the adrenoceptor agonist isoprenaline stimulates pump activity in a PKA-dependent manner when intracellular calcium is clamped to physiological or slightly supra-physiological concentrations [68, 72], but isoprenaline inhibits the pump in a PKA-dependent manner when intracellular calcium is clamped to sub-physiological concentrations [70, 72]. In general laboratories reporting pump inhibition by PKA buffer intracellular calcium below 100 nM (for example [69]), while those reporting pump activation by PKA do not buffer calcium (for example [39, 61, 63])—which may account for the contradictory results between different investigators. This effect of calcium buffering on PKA-mediated pump activation is consistent with our observation that the elevated sodium affinity of the pump in PLM knockout myocytes is dependent on pipette calcium, this effect being lost when calcium is buffered below around 10 nM [73]. Indeed, calcium is also reported to modify the effect of PKA activation on pump activity in an identical fashion in tissues other than the heart [74]. Whether this effect of calcium is a biophysical quirk or a genuine physiological means to switch the pump from a PKA-inhibitable to a PKA-activatable state is not known. Calcium activation of Ca-sensitive enzymes such as PKC may play a role, however in the intact heart, the average intracellular calcium, which is dependent on heart rate and adrenergic tone, will always normally exceed the 10–150 nM cut-off above which PKA stimulates the pump [72, 75]. This would suggest that PKA inhibition of the pump is of limited biological relevance in cardiac muscle, but is perhaps important in cell types where intracellular calcium is lower.

#### Protein kinase C

In some tissues there is convincing evidence that PKC phosphorylation of the pump α subunit is a signal for pump internalization and degradation (for example [76–78]), but this does not appear to be the case in the heart. Again, there is disagreement with respect to the functional effect of PKC activation on the pump in the heart, with both activation [61, 79–81] and inhibition [82–84] reported. Again, it has been suggested that intracellular calcium determines the functional effect of PKC on the pump in the kidney [74], and this may also prove to be the case in the heart. However, an additional level of complication is the multiplicity of PKC isoform expression in the heart—with at least PKCα, δ and ε found expressed [61], and therefore the potential for PKC isoform-specific effects on pump activity. That calcium-sensitive PKCs activate the pump is suggested by the observation that simply increasing extracellular calcium is sufficient to increase pump *V*_max_, presumably through an effect on these PKCs [85].

#### Nitric oxide

There is near-universal agreement that nitric oxide (NO) stimulates the cardiac Na pump [86–89], although it is reported to inhibit the pump in other tissues, notably the kidney [90, 91]. NO synthesis in cardiac muscle may be activated by natriuretic peptide receptors [86] and β3 adrenoceptors [89], and has also been reported to be controlled by heart rate [92, 93], although the impact of the latter on pump activity remains uninvestigated to date. It is, however, noteworthy that all tissues in which NO is reported to stimulate the pump either express PLM, or the associated FXYD accessory protein is unknown. This raises the possibility that PLM is required for the stimulatory effects of NO on the pump [94].

### Phospholemman the kinase target

Phospholemman (PLM) was first identified as an abundant phosphoprotein in the cardiac sarcolemma in 1985 [95], and it was quickly recognized to be the principal sarcolemmal substrate for both PKA and PKC in the heart [95, 96]. Thereafter, it was proposed to form an anion conductance in its own right [97, 98], but remained something of an orphan. Indeed, 13 years after its discovery, its role in the heart remained something of a mystery: *As a major target for hormone*-*stimulated phosphorylation in the heart, the physiological function of phospholemman is likely to be an important one* [99]: it was clearly important, but just what did it do? The identification of the FXYD family of pump regulators [11] of which PLM is a member gave us our answer: PLM associates with the Na pump in the heart [16, 59, 100–102], and modifies its transport properties (see below).

**Fig. 1:**
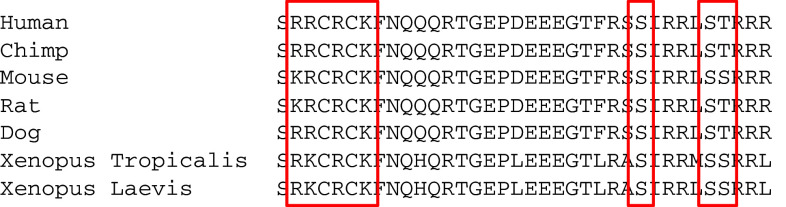
Sequence alignment of phospholemman from different species. The phosphorylation sites and palmitoylation/glutathionylation sites plus surrounding residues are conserved across vertebrate classes

PLM (FXYD1) is unique in the FXYD family in possessing phosphorylation sites in its carboxyl terminus that are conserved across all vertebrate sequences cloned to date (Fig. 1). PLM is phosphorylated at S63, S68, and S/T69 by PKC, and at S68 by PKA [61, 103]. PLM S63 has also been reported to be a substrate for NIMA kinase [104], although there is no known functional connection between this kinase and the pump.

There is not universal agreement over the exact functional effect of PLM on the cardiac Na pump. In part, this stems from the variety of experimental models and approaches. These range from biochemists requiring a precise kinetic analysis of what each part of the PLM molecule does to the pump (for example [105]), to physiologists who are perhaps more concerned to find out what happens to the pump when the cell is exposed to agonist X, Y, or Z (for example [39]). As will be discussed, not all observations from different laboratories can be reconciled, but a consensus on the functional role of PLM has emerged in recent years.

Unphosphorylated PLM inhibits the cardiac Na pump. The exact nature of the inhibitory effect has been reported to be via a reduction in sodium affinity with no alteration in maximum transport rate (in voltage-clamped and fluorescent sodium indicator SBFI-loaded ventricular myocytes [39, 59, 81, 106] or following reconstitution of recombinant αβ with recombinant PLM [105]), a suppression of pump-maximum transport rate (in voltage-clamped ventricular myocytes [61–63, 107]), or α subunit isoform-specific effects on both [108] (see below). There are also reports that recombinant PLM activates the pump upon reconstitution [109, 110], which will also be considered below (“Oxidant modification as a reversible regulator of the pump”).

The interaction between PLM and the α subunit of the pump has been visualized as co-immunoprecipitation [16, 101], intermolecular crosslinking [101], and intermolecular FRET [102]. Early studies on the relationship between these proteins noted that they remained associated whether or not PLM was phosphorylated, but that phosphorylation appeared to alter their relative alignment such that the α subunit was crosslinked less efficiently to phosphorylated PLM [101]. These observations were confirmed and refined by experiments that demonstrated that PLM-YFP and α1-CFP exhibited significant (20 %) FRET, and that this FRET was almost abolished when PLM was phosphorylated [102]. Hence, the PLM carboxyl terminus (where the YFP fluorophore was fused) is in close proximity (<9 nm) to the pump α subunit, and this distance is significantly increased when PLM is phosphorylated.

#### Phospholemman and PKA

The kinase signaling pathways discussed in “Adrenergic signaling pathways” that lead to Na pump activation are now widely acknowledged to terminate at PLM. Phosphorylation of PLM increases pump activity by relieving the inhibitory effect of PLM on the pump [39, 59], and in some models actually increases *V*_max_ of the pump [62, 81, 101]. The most convincing experiments in this regard come from the PLM knockout mouse, which has a relatively moderate cardiac phenotype (mild left ventricular hypertrophy and depressed in vivo cardiac function) consistent with elevated pump activity unloading the SR via an effect on NCX [111]. In myocytes isolated from PLM KO animals and their WT littermates, the sodium affinity of the pump (assessed by simultaneous measurement of intracellular sodium and pump current) is higher in the KO, because the inhibitory effect of unphosphorylated PLM is lost. Stimulation of PKA via β-adrenoceptor activation is without effect in the KO, but increases pump activity in the wild-type by increasing the sodium affinity—such that the pump activity in wild-type myocytes stimulated with isoprenaline resembles that in the KO [39]. Hence, it was proposed that the relationship between PLM and the pump is analogous to the relationship between the SR calcium ATPase SERCA and its regulatory protein phospholamban (PLB) [39]: unphosphorylated accessory protein inhibits the ATPase by reducing its affinity for the transported cation, and this inhibition is relieved by phosphorylation. Although there is strong homology between PLM and PLB around the phosphorylation sites, they are evolutionarily unrelated proteins, with opposite transmembrane orientations. Nevertheless, they bind and regulate two closely related ion pumps in SERCA and the Na pump.

Subsequent research has suggested that the relationship between PLM and the Na pump is possibly more complex than that between PLB and SERCA. PLB is a substrate for PKA (and S16) and Cam kinase (at T17), with essentially identical effects on SERCA activity of phosphorylation at either site [112]. The analogous residues in PLM are S68 and S/T69, but PLM may also be phosphorylated at S63 by PKC (discussed below). Several investigators have reported effects of phosphorylated PLM on Na pump maximum transport rate in ventricular myocytes [61–63, 107]: PLB has never been observed to alter the *V*_max_ of SERCA.

#### Basal phosphorylation of phospholemman

One complication in the investigations into the relationship between PLM and the Na pump is the relatively high basal phosphorylation of PLM in ventricular muscle. In rat ventricular myocytes, ~30 % of PLM is phosphorylated at S68 and ~50 % is phosphorylated at S63 (such that ~40 % of PLM is phosphorylated at neither residue, and ~30 % is phosphorylated at both) [61]. This basal phosphorylation is the result of PKC activity, and underlying it is a rapid turnover of phosphorylation and dephosphorylation: when PKC isoforms are acutely inhibited, PLM is dephosphorylated at both sites with a half-life of 2–3 min [61]. The consequences of this high basal phosphorylation are twofold. Firstly, agonist-induced phosphorylation of PLM in rat ventricular muscle generally elicits only a two- to threefold increase in phosphorylation of S63 or S68, (although the third PKC site S/T69 is essentially unphosphorylated in the basal state and its phosphorylation is therefore very significantly elevated by PKC agonists [61]). While this has not been formally investigated in other species, PKA and PKC activation in mouse ventricular myocytes also elicits only up to twofold increases in phosphorylation of PLM S63, and up to fivefold increases in phosphorylation of PLM S68 [81]. Secondly, the comparison of pump activities between PLM wild-type and KO myocytes compares a mixture of phosphorylated and unphosphorylated PLM in the wild-type, to the absence of PLM in the KO. Since one of the factors likely to influence PLM phosphorylation is the adrenergic state of the heart when myocytes are prepared, “resting” PLM phosphorylation is likely to vary from laboratory to laboratory, and even from day to day in the same laboratory. In addition, and clearly related, is the observation that basal phosphorylation is extremely sensitive to the resting cellular calcium load and hence is directly related to the “quality” of the isolated myocytes. This basal PLM phosphorylation can be substantially reduced either by lowering extracellular calcium or by treating cells with bisindolylmaleimide implicating the calcium-activated PKCs in this basal tone [61]. Consistent with this, we observe lower basal phosphorylation when PLM is expressed in cultured cells, or when cultured myocytes have been allowed to recover from the stresses of isolation. These cells are accordingly more responsive in terms of fold changes in phosphorylation following agonist application (for example see [15, 61]).

The relative contributions of basally phosphorylated and unphosphorylated PLM to pump regulation in wild-type myocytes was addressed by Pavlovic et al. [62]. A peptide corresponding to the final 19 amino acids of rat PLM (including all phosphorylation sites) was applied to the intracellular face of myocytes from PLM wild-type and KO animals using the whole cell configuration of the patch clamp. This peptide reconstituted with and inhibited the pump in both wild-type and KO myocytes—revealing the functional contribution of basally phosphorylated PLM to pump activity. When peptide phosphorylated by PKA at S68 was applied, pump activity was stimulated in wild-type cells (revealing the functional contribution of unphosphorylated PLM), but also surprisingly in KO cells. Hence, while confirming the concept that unphosphorylated PLM is an endogenous inhibitor of the pump, this investigation raised the possibility that phosphorylated PLM actively stimulates the pump rather than simply relieving an inhibition. The ability to activate the pump must therefore lie within the PLM phosphorylation sites. Reconstitution experiments and functional evaluation of PLM/FXYD4 chimeras indicate that some inhibitory effect on the pump (through a reduction in sodium affinity) is mediated by the PLM transmembrane domain [105]. Therefore two regions of PLM inhibit the pump: the transmembrane domain and the intracellular region [62]. Whether wild-type, endogenous PLM activates the pump in cardiac muscle to the same extent as a peptide applied to KO cells through the patch pipette is doubtful. Not only will full-length PLM transmembrane domain exert an inhibitory effect on pump sodium affinity, but phosphorylation has also recently been found to promote palmitoylation of PLM, which itself inhibits the pump (discussed below) [15].

#### Phospholemman and PKC: isoform-specific regulation?

Na pump activation by PKC in the heart also requires PLM [81]. In mouse ventricular myocytes, phosphorylation of PLM by PKC substantially increases pump *V*_max_ [81], with no change in sodium affinity in some reports [81], and an increase in others [59]. The effects of PKA and PKC activation turn out to be additive, both in terms of phosphorylation of PLM and functional effect on the pump. So activating PKA after PKC elicits additional phosphorylation of PLM at S68, and increases pump sodium affinity (against a background of already-increased *V*_max_), and activating PKC after PKA causes further phosphorylation of S68 and increases pump *V*_max_ [81]. Taken together, these studies suggest that in murine ventricular myocytes, PKA and PKC have access to different pools of PLM, and the functional effect of phosphorylating these pools is different. While this *may* reflect a pump isoform-specific effect of PLM phosphorylation on the pump, in the mouse, almost 90 % of pump is α1-containing [19]. Hence, although there is functional evidence to support the notion that PKA is functionally linked to α1-containing pumps only [19, 113], it is unlikely that the exclusive linking of PKC to an α2-containing pool that is only ~10 % of total myocyte pump could account for the 60 % increase in pump *V*_max_ observed when PKC is activated in the mouse ventricular myocytes [81].

That PKA and PKC are functionally linked to different isoforms of the catalytic subunit of the pump in cardiac muscle was first proposed even before the role of PLM in regulating the pump had been discovered [114]. In the* Xenopus* oocyte expression system, PKA phosphorylation of PLM at S68 increases the sodium affinity of both α1 and α2-containing pumps, whereas PKC phosphorylation of PLM increases the turnover rate only of α2 containing pump (to a level above the activity of pump expressed without PLM) [108], closely paralleling the results described above in mouse [81]. The functional effect of both PKA and PKC activation on both pump isoforms in this oocyte system requires phosphorylation of PLM S68 [108]. Importantly, the fact that this result was obtained in a simple oocyte expression system would seem to rule out any role for differential kinase targeting of PKA to an α1-only pool and PKC to an α2-only pool of pump that might account for observations made in mouse ventricular myocytes [81]. Instead, this implies that while both α1 and α2-containing pumps have an identical capability to sense phosphorylation of PLM S68 (leading to a change in sodium affinity), α2-containing pumps are uniquely able to sense, additionally, phosphorylation at PLM S63 (and in all likelihood PLM S/T69, which was not described at the time the study was conducted). The impact of this additional phosphorylation event is to increase the turnover rate of α2-containing pumps above that observed in the absence of PLM. The molecular basis of this insensitivity of the α1 subunit to phosphorylation of PLM S63 remains to be determined: beyond the transmembrane domain [105], the sites of interaction between PLM and pump α subunits have not been defined. Given that the increase in α2 *V*_max_ induced by PKC phosphorylation is abolished by mutation of PLM S68 [108], it is likely to be an electrostatic effect of multiple phosphorylations in the PLM C terminus, rather than a specific effect of PLM S63 phosphorylation per se. Such a notion is supported by the fact that phosphorylation-induced changes in FRET between PLM-YFP and CFP-α1 and PLM-YFP and CFP-α2 are indistinguishable [59]. That said, the phosphorylation-induced reduction in FRET between PLM-YFP and α2-CFP is perhaps incompatible with the concept of an α2 subunit-specific activation (rather than disinhibition) of the pump by S63 phosphorylated PLM.

**Fig. 2:**
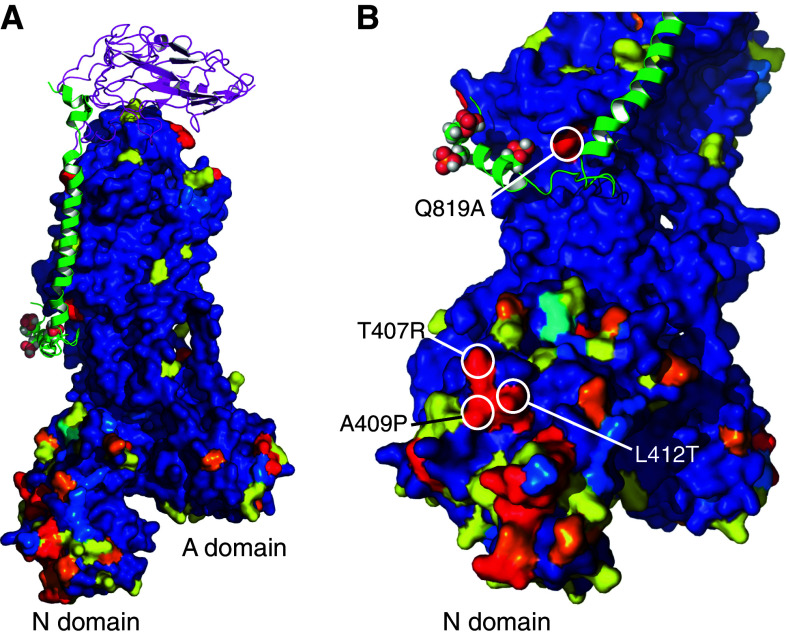
Sequence divergence between α1 and α2 subunits as a basis of differential regulation by PLM? Na pump α1 and α2 sequences from the species indicated were aligned with Clustal (for full alignment, see Supplement 1). A heat map was generated using the porcine crystal structure (3B8E.pdb [3]) to indicate positions of surface conservation and divergence between α subunits using a 1–5 scale (annotated on the Clustal alignment). The β1 subunit is shown in magenta, and PLM (phosphorylated at S63, S68, and T69) is shown in green, positioned according to [15]. *Color coding* of the α subunit is as follows: (1) *Dark Blue* 85+ % conserved (where both α1 and α2 are the same, allowing only one outlier in both groups). (2) *Light blue* either no consensus for α1 and α2, or used if α1 and α2 cannot be discriminated significantly. (3) *Yellow* conservative change, represented by ‘;’ in the Clustal alignment. Also used even if there is a single outlier in one group. (4) *Orange* moderate change, represented by ‘.’ in the Clustal alignment. (5) *Red* major change. Also used even if a single outlier is present. If multiple outliers are present, this is downgraded to yellow. **a** Surface divergence between α1 and α2 is particularly notable in the N domain. **b** Major changes between α1 and α2 that may be influenced by phosphorylation of PLM are highlighted

It is perhaps significant that there are notable sequence differences between α1 and α2 subunits in a region of the N domain that may be capable of interacting with PLM, which might explain the isoform-specific effects of PLM phosphorylation. Figure 2 models the regions of divergence between α1 and α2 subunits in mammals on the crystal structure of the porcine pump. Blue indicates little or no divergence, yellow conservative changes, orange moderate changes, and red major changes in sequence between the isoforms. No Na pump crystal structure has yet resolved a significant portion of the intracellular region of the associated FXYD protein, which implies significant mobility for this region. The flexible linker between PLM helix 3 (H3) and helix 4 (H4 contains the phosphorylation sites) may allow H4 to adopt multiple positions relative to the α subunit depending on its phosphorylation state: in other words, the PLM NMR structure may not accurately represent the structure it adopts when complexed with the pump. This is emphasized by the lack of sequence difference between α1 and α2 around the position that PLM H4 is modeled based on its NMR structure (Fig. 2b, save for Q819 in α1 to A in α2), which is not concordant with the specific activation of α2 by PKC phosphorylation of PLM. However, regions of the N domain that are orientated towards PLM in the E2 crystal structure do show divergence between α1 and α2: in particular T407R, A409P, L412T, and Q521I in a surface groove of the N domain facing PLM (Fig. 2b). The presence of a positively charged arginine in position 407 of α2, and removal of the nearby hydrophobic leucine (in position 412 of α1), may be of particular significance for transducing the effect of multiple phosphorylations of PLM to the α2-containing pump if the H3/H4 linker in PLM is able to flex sufficiently to allow H4 to interact with the α2 N domain in either E2 or the as yet uncrystallized E1 state. Since the rate-limiting step in the reaction cycle of the pump is the E2 to E1 transition [115], the effect of PLM phosphorylation on α2 pump *V*_max_ must reflect an increase in the rate of this partial reaction. Doubtless the mechanism underlying this will become clearer when more structural information is available.

The elegant transgenic model in which the ouabain affinities of Na pump α1 and α2 subunits are swapped (SWAP mice [18]) has allowed the relative contributions of PKA and PKC-mediated phosphorylation of PLM to regulation of α1- and α2-containing pumps in cardiac myocytes to be assessed. The ouabain sensitivity of mouse α1-containing pump is elevated by the mutations R111Q and D122N in the first extracellular loop: Q111 and N122 are found in the high affinity human and sheep α1 isoforms and confer sensitivity to cardiac glycosides [116, 117]. The ouabain sensitivity of α2-containing pumps is reduced by mutations L111R and N122D in the same position. Hence the ouabain sensitivities of the cardiac pumps are swapped without altering isoform distribution or enzymatic activity [18]. In the wild-type mouse, the dominant α1 isoform is ouabain-resistant, and α2 is ouabain-sensitive, such that 10–20 μM ouabain will block essentially all α2-containing pumps, while leaving ~90 % of the α1-containing pumps active. The same low ouabain concentration specifically blocks α1-containing pumps only in SWAP mice, meaning the relative contributions and regulation of α1 and α2 can be determined in SWAP and wild-type mice, respectively [59]. Much as was reported in oocytes [108], phosphorylation of PLM by PKA increases the sodium affinity of both α1 and α2 isoforms. Phosphorylation of PLM by PKC also elevates sodium affinity of both isoforms, but again only elevates the *V*_max_ of α2-containing pumps [59]. For now, the basis of the α2-specific effect of PLM remains unresolved. Apart from the sequence differences discussed above, it may be relevant that there are clear biochemical differences between α1β1 and α2β1 pumps in terms of thermal stability and interaction with phospholipids [118] that could account for this.

#### Phospholemman and phosphatases

Relatively little research has been conducted to date on the pathways leading to PLM dephosphorylation. Shortly after the cloning of PLM, adenosine receptor agonists were reported to attenuate adrenoceptor agonist-induced phosphorylation of PLM independent of cellular cAMP [119]. Subsequent work found that PLM is a substrate for both PP1 and PP2A, and it therefore provides a functional link between both these phosphatases and the Na pump [120]. More recently it has been shown that PLM S68 (and possibly T69) are substrates for PP1, while S63 is probably a PP2A substrate [121]. Moreover, phosphorylation of S68 is regulated by the PP1 inhibitor inhibitor-1: intracellular application of an inhibitor-1-derived peptide, or overexpression of inhibitor-1 leads to enhanced phosphorylation of PLM S68 and increased Na pump currents [121]. In failing human hearts, PP1 hyperactivity may contribute to impaired β-adrenoceptor responsiveness [122], and this reduced phosphorylation of PLM at S68 [121]. Under-phosphorylation of PLM in failing cardiac tissue leading to reduced Na pump activity may be a causal event in the well-characterized elevation of intracellular sodium in human heart failure [123, 124]. Hence, the PLM dephosphorylation pathways may be a ripe therapeutic target in the management of elevated intracellular sodium in the failing heart.

#### The functional role of phospholemman phosphorylation

In the context of adrenoceptor activation increasing myocardial contractility, it is pertinent to ask why hearts need PLM. On the face of it, enhanced Na pump activity, by increasing the driving force for calcium efflux through NCX, will tend to oppose the positive inotropy achieved through activation of L-type calcium channels, SERCA, and the ryanodine receptor by PKA. Genetic deletion of PLM slightly reduces cardiac contractility in vivo, although this is partly offset by a (possibly adaptive) reduction in pump subunit expression [111]. It turns out that the small price paid in terms of reduced inotropy when phosphorylated PLM activates the pump is more than balanced by the protective effect of PLM phosphorylation [125]. In myocytes from PLM KO animals, an increase in stimulation frequency plus β-adrenoceptor activation with isoprenaline causes a larger rise in intracellular sodium, greater SR calcium content, and bigger calcium transients than in myocytes from wild-type animals from essentially identical baselines in unstimulated cells [125]. However, the overloading of the SR with calcium leads to more spontaneous calcium transients and hence arrhythmias in PLM KO myocytes. Therefore PLM phosphorylation is a protective event in the heart. By limiting sodium (and therefore calcium overload), PLM protects the heart from arrhythmias and contractile disturbances linked to adrenoceptor activation. There is a clear parallel to the human condition catecholamine-induced polymorphic ventricular tachycardia (CPVT), but to date, no human mutations of PLM associated with CPVT have been described. The clinical significance of the PLM/Na pump relationship may be confined to the under-phosphorylation of PLM described in “Phospholemman and phosphatases”.

#### Phospholemman palmitoylation: the new kid on the block

*S*-palmitoylation is the reversible covalent post-translational attachment of the fatty acid palmitic acid to the thiol group of cysteine, via an acyl-thioester linkage [126]. In recent years, protein *S*-palmitoylation has emerged as an important and common post-translational modification in a variety of tissues [127]. Protein *S*-palmitoylation is catalyzed by palmitoyl acyltransferases, reversed by protein thioesterases, and occurs dynamically and reversibly throughout the secretory pathway in a manner analogous to protein phosphorylation [126]. Many different classes of protein have been identified as targets for palmitoylation, including G-proteins [128, 129], ion channels [130], transporters [131], receptors [132], and protein kinases [133, 134]. Of particular relevance to this review, palmitoylation can control ion channel/transporter activity, stability, or subcellular localization [127, 135]: it has the potential to induce substantial changes in the secondary structure and therefore function of intracellular loops through their recruitment to the inner surface of the membrane bilayer.

PLM is palmitoylated at two intracellular cysteines, C40 and C42, just beyond the transmembrane domain [15]. Notably, these cysteines are conserved across species, but also one or both cysteines are found in analogous positions throughout the FXYD family: FXYD2, 5 and 7 have one, and FXYD3, 4 and 6 have two [136], and all are predicted to be palmitoylated [15], meaning FXYD protein palmitoylation may be a universal means to regulate the pump. Unpalmitoylatable mutant PLM is degraded with a shorter half life than wild-type PLM in transiently transfected cells, but the principal functional effect of PLM palmitoylation is inhibition of the Na pump [15]. The inhibitory effect of PLM on the pump is abolished following application of the pharmacological inhibitor of palmitoyl acyltransferases 2-bromopalmitate. Indeed, given the relatively modest effect of this inhibitor on PLM palmitoylation, it is possible that palmitoylation switches PLM from a pump activator to an inhibitor.

**Fig. 3:**
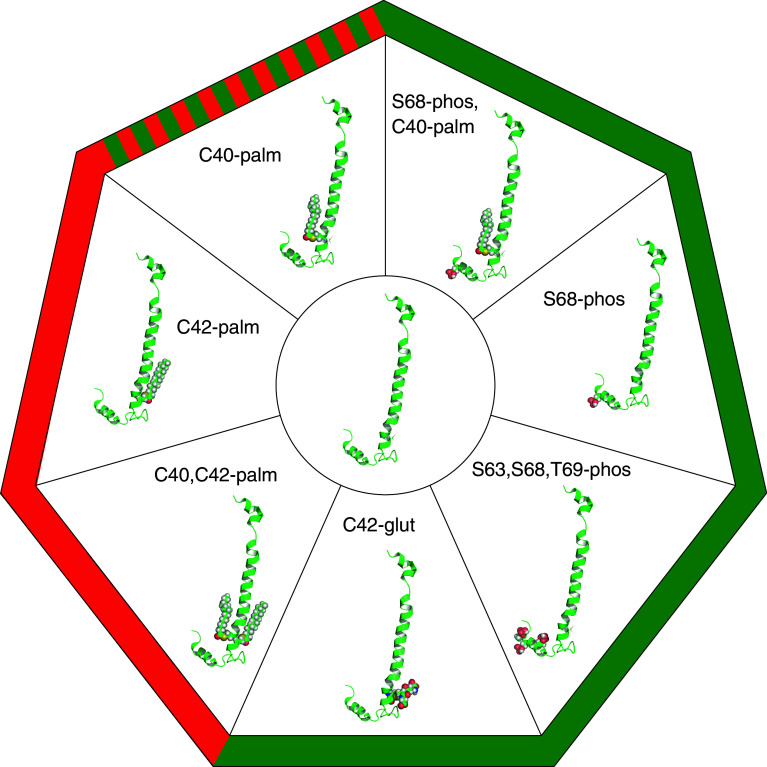
The many faces of phospholemman. The NMR structure of PLM (2JO1.pdb [137, 138]) is in the center, and the post-translational modifications of PLM discussed in the text are shown. The functional effect of each modification on pump activity (compared to unmodified PLM) is indicated by *green* (for activation), *red* (for inhibition), or both *red* and *green* where this remains to be determined

**Fig. 4:**
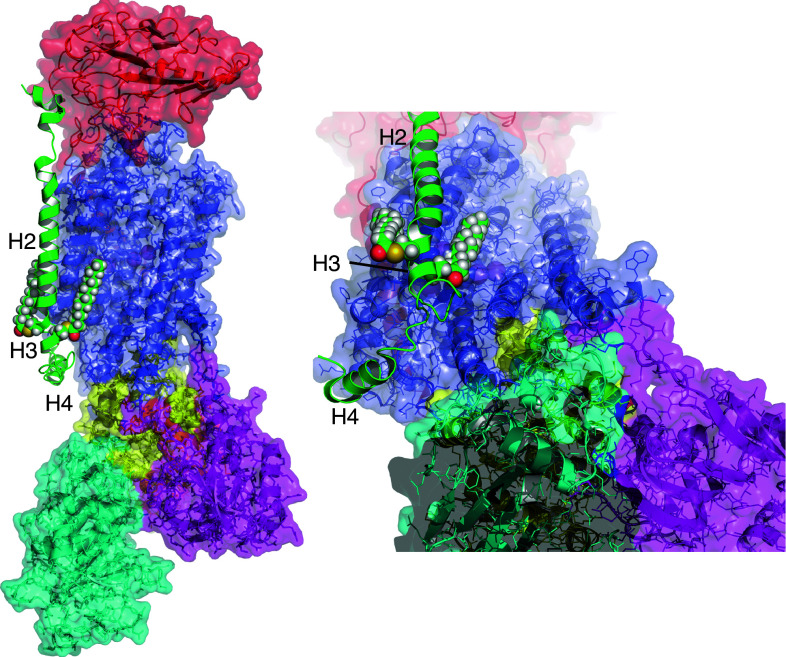
Position of the palmitoylation sites of phospholemman relative to the α subunit. PLM is shown in *green*, α subunit transmembrane region in *blue*, N domain in* cyan*, P domain in *yellow*, and A domain in *purple*. The β subunit is *red*. Sodium (*purple spheres*) is shown in its proposed binding sites (reviewed in [6]). Helices H2, H3, and H4 of PLM are labeled. C42 of PLM is orientated towards the α subunit, and C40 away, meaning palmitoylation of C42 may pull PLM H3 across the intracellular mouth of a sodium-binding site in the α subunit. Conversely, palmitoylation of C40 would oppose such a movement by pulling H3 in the opposite direction

Although the palmitoyl acyltransferase that palmitoylates PLM is yet to be identified, one important regulator of PLM palmitoylation is its phosphorylation status. Paradoxically, phosphorylation of PLM at S68 by PKA increases PLM palmitoylation [15]. Hence, one post-translational modification of PLM that activates the Na pump promotes a second that inhibits it. The molecular basis of phosphorylation promoting palmitoylation can probably be explained by reference to the NMR structures of unphosphorylated and S68 phosphorylated PLM [137, 138] (Fig. 3). S68 phosphorylation of PLM increases the mobility of PLM helix 4 relative to unphosphorylated PLM, without inducing major changes in the overall structure of the protein. This probably increases the accessibility of the cysteines in PLM helix 3 to the palmitoyl acyltransferase enzyme(s) that palmitoylate PLM. As for the physiological and functional significance of enhanced PLM palmitoylation following PKA activation, this remains to be seen. Site-specific reagents to distinguish which cysteine in PLM is palmitoylated following S68 phosphorylation of PLM do not exist (nor do they for other palmitoylation sites in other proteins). Molecular models of the PLM/Na pump complex (Fig. 4) suggest PLM C42 could mediate the inhibitory effect of PLM palmitoylation on the pump, as the side chain of this amino acid is orientated towards the pump α subunit, and C40 is orientated away. Palmitoylation of C42 (with incorporation of the palmitate into the lipid bilayer) may pull PLM H3 across the intracellular mouth of a sodium-binding site in the α subunit in order to inhibit the pump. Conversely, palmitoylation of C40 on the opposite side of H3 would oppose such a movement by pulling H3 in the opposite direction. This raises the possibility that while the overall effect of PLM palmitoylation on the pump is inhibitory, the individual palmitoylation sites may have opposing effects on pump activity through their reorientating effects on PLM H3 (Fig. 4).

#### Other roles of phospholemman

Although beyond the scope of this review, it is important to note that several other functional roles are ascribed to PLM in the heart. Phosphorylation of PLM at S68 is associated with inhibition of NCX [139, 140]. It is proposed that NCX inhibition is necessary in the context of Na pump activation by PLM to prevent the enhanced sodium gradient driving NCX to unload the SR of calcium following adrenergic stimulation [140]. In addition, PLM modulates L-type calcium channel gating when expressed with Cav1.2 in heterologous cells: it slows activation and deactivation, and increases the rate of voltage-dependent inactivation [141] via an effect of the extracellular FXYD motif [142]. It remains to be determined if endogenous PLM associates with and regulates L-type calcium channels in the heart: it is possible these effects simply reflect the abnormal interaction of an over-expressed membrane-spanning protein with a sarcolemmal ion channel.

Although the original proposal [98] that PLM oligomerizes to form an ion channel and is involved in regulation of cell volume is not now widely favored, convincing experimental evidence suggests that PLM does oligomerize [102, 143], at least following expression in heterologous cells. PLM-YFP and PLM-CFP exhibit significant FRET with each other, which is increased following PLM phosphorylation [102], or by phosphomimetic mutations of PLM [143]. Peptides corresponding to the transmembrane domain of PLM form stable homo-tetramers [144, 145] and progressive acceptor photobleaching experiments also suggest that the PLM–PLM oligomer is composed of three or more molecules [59]. The physiological significance of this in cardiac muscle remains to be determined. The analogy to PLB and SERCA may be relevant: PLB monomers inhibit SERCA [146], while PLB pentamers are thought to be inactive [147]. PLM may also exist in multiple states in cardiac muscle: in a 1:1 complex with the pump [59], and in homo-oligomers—possibly a storage pool. If some Na pump in the heart is PLM-free (which has been suggested [62]) the storage pool would represent a means to increase the ‘responsiveness’ of the pump to adrenergic stimulation, by as-yet unidentified means.

### Oxidant modification as a reversible regulator of the pump

In contrast to the apparent contradictions in the literature regarding the effect of PKA and PKC agonists on the cardiac pump, the exquisite sensitivity of Na pump activity in the heart to oxidant stress is widely accepted. Shattock and Matsuura [38] investigated the direct effect of free-radical induced stress on the pump in voltage-clamped rabbit ventricular myocytes. Their work indicated that pump current was reduced by approximately 50 % at all membrane potentials after a 10-min exposure to photoactivated rose-bengal (a singlet oxygen and superoxide generator). Subsequent work found pump activity was significantly reduced by intracellular application of thiol-modifying reagents, or depletion of cellular glutathione [148], confirming a functional link between pump activity and protein sulfhydryl status.

Oxidant modification and regulation of the pump has also been the subject of more recent investigation. Glutathionylation is the reversible conjugation of the tripeptide glutathione to protein cysteines in a mixed disulfide. Many classes of protein have been found to be regulated by glutathionylation, including metabolic enzymes such as glyceraldehyde-3-phosphate dehydrogenase [149], kinases including PKA [150] and PKC [151], G proteins such as H-Ras [152] and ion transporters and pumps such as the ryanodine receptor [153] and SERCA [154]. Like phosphorylation and palmitoylation, glutathionylation allows dynamic, reversible post-translational regulation of all manner of signaling and metabolic pathways [155]. Glutathionylation is no longer considered solely to be a consequence of oxidative stress, and occurs under physiological conditions in the absence of overt oxidative burden [156]. Unlike phosphorylation and palmitoylation, glutathionylation is *generally* non-enzymatic, and occurs by direct reaction between oxidized/modified protein sulfhydryls and cellular glutathione, direct reaction between protein sulfhydryls and modified (e.g., nitrosylated) glutathione, disulfide exchange between protein sulfhydryls and glutathionylated proteins, or direct reaction between protein sulfhydryls and glutathione in the presence of oxidants [155]. The lack of enzymes to catalyze glutathionylation does not result in a lack of specificity for this modification, as the vast majority of cysteine sulfhydryls are not susceptible to glutathionylation because their pKa is above 8.0, such that they remain protonated and hence non-reactive at physiological pH. Redox sensitive cysteines that are susceptible to glutathionylation may be deprotonated to the thiolate anion at physiological pH as a result of a favorable local environment for that particular cysteine, usually achieved by the presence of positively charged amino acids nearby to receive the proton. Glutathionylation is reversed enzymatically by glutaredoxins, thioredoxins and sulfiredoxin, and can be removed non-enzymatically by disulphide exchange with glutathione [155].

The α subunit of the cardiac Na pump has recently been reported to be glutathionylated to regulate its activity [157]. Exposure of purified Na pump to oxidized glutathione causes glutathionylation of conserved cysteines in the α subunit actuator and nucleotide binding domains. Glutathionylation of the cardiac pump occurs in the basal state, and is promoted during cardiac hypoxia, causing profound pump inhibition. Interestingly, ATP binding and α subunit glutathionylation are competitive: glutathionylation only occurs at ATP concentrations below 0.5 mM, and ATP is modeled to be unable to bind to the glutathionylated α subunit [157], which accounts for the inhibitory effect of glutathionylation on the pump. Pump α2 subunit is more sensitive to inhibition by glutathionylation than α1, which may be relevant in the light of the different physiological roles for these subunits discussed in “Introduction” and “Pump regulation by the prevailing cellular environment”. It is proposed that rapid, reversible pump inhibition by glutathionylation during depletion of cellular ATP (for example during cardiac ischemia or hypoxia) is a means to protect the pump α subunit against irreversible oxidation, and protect the cell against ATP depletion by the pump [157]. This protection of the pump and energy expenditure, however, comes at a price—the inhibition of the pump and the associated elevation of intracellular sodium that is known to occur rapidly after the onset of ischemia [158–160].

The Na pump in the heart is also reported to be regulated by glutathionylation of its β rather than α subunit [161]. The cardiac β1 subunit is found glutathionylated in unstimulated myocytes, and exogenous oxidants such as peroxynitrite or hydrogen peroxide promote additional glutathionylation of the β1 subunit when applied to intact cardiac myocytes [161]. A similar result is observed following activation of NADPH oxidase, which is reported to colocalize with the cardiac pump and mediate angiotensin II-induced pump inhibition via a PKCε dependent pathway [84]. The functional effect of β1 subunit glutathionylation is to destabilize the interaction between cardiac α and β subunits, and decrease the *V*_max_ of the pump through a reduction of the rate constant for the E2 to E1 transition in the reaction cycle [161]. The cysteine residue within the β1 subunit that becomes glutathionylated is in position 46, which is perhaps surprising given this amino acid is well within the membrane spanning domain of the β1 subunit in the crystal structure of the pump in the E2 state [4]. Recent work has shed light on this however: when the E1 state of the pump is stabilized, C46 of β1 becomes both more susceptible to glutathionylation in the presence of oxidants, indicating the molecular rearrangement during transition from E2 to E1 is sufficient to expose this site to the cytosol [162]. Ouabain, by stabilizing the pump in the E2 conformation, reduces the susceptibility of the β1 subunit to glutathionylation [162].

A similar signaling mechanism has been proposed to mediate *inhibition* of the cardiac pump by PKA [69]. In rabbit cardiomyocytes, PKA stimulation with the adenylyl cyclase activator forskolin is reported to activate NAPDH oxidase in a PKCε-dependent manner, which leads to glutathionylation of the cardiac β1 subunit and pump inhibition. Herein a remarkable contradiction emerges in the regulation of the Na pump by PKA and PKC in the heart. The majority of researchers report PLM-dependent activation of the pump by both kinases [39, 59, 61–63, 81, 101, 108, 121, 125], but some describe PLM-independent, NADPH oxidase-dependent inhibition of the pump by the same kinases over essentially identical timescales [69, 84, 161]. Unless the effect of calcium buffering discussed above (“Protein kinase A”) can really account for these differences, it is certainly difficult to reconcile such contradictory observations. It is also difficult to conceive how it might be useful for the heart to have the same pathways mediating such contradictory effects on pump activity in the same cell type. A key determinant may be the presence of PLM, since this has been reported to relieve the inhibition of the pump caused by glutathionylation of its β1 subunit [110] (discussed in “Phospholemman: integrating multiple post-translational modifications?” below). This may render PLM-containing pumps resistant to oxidative inhibition, and at the same time activatable by protein kinases. Doubtless such incongruities will be the topic of future research and debate.

A signaling pathway through the β3-adrenoceptor is also linked to regulation of the cardiac pump. Activation of β3 receptors leads to pump stimulation in an NO and guanylate cyclase-dependent manner, through a reduction in the glutathionylation of the pump β1 subunit [89]. Hence, cellular mechanisms exist to enhance de-glutathionylation as well as glutathionylation. The molecular basis by which NO might reduce β1 subunit glutathionylation remains unclear. It may be relevant that neither α nor β subunits of the pump are likely targets of cGMP signaling pathways, whereas PLM is [163].

### Phospholemman: integrating multiple post-translational modifications?

Experiments in which recombinant FXYD proteins are reconstituted with the Na pump have recently suggested that recombinant PLM is capable of activating the pump [109, 110], although some investigators report the opposite phenomenon in very similar model systems (for example, see [105]). A component of this activation could be explained by the stabilizing effect of PLM on the pump complex [118, 164], however other mechanisms have also been invoked. PLM is proposed to be glutathionylated to relieve oxidant-induced inhibition of the pump following glutathionylation of its β1 subunit [110]. PLM glutathionylation is promoted by the same signaling pathways that promote β1 subunit glutathionylation, but remarkably PLM glutathionylation is suggested to be downstream of β1 subunit glutathionylation. That is, rather than being glutathionylated as an alternative to β1 subunit C46, the presence of PLM facilitates de-glutathionylation of C46 of β1, with concomitant glutathionylation of PLM [110]. PLM and the β1 subunit of the pump are a considerable distance apart in the current crystal structures in the E2 state [3, 4]. Glutathionylation occurs at PLM C42, and requires the adjacent amino acid K43, presumably to provide a locally positively charged environment for the deprotonation of C42. Thus the presence of PLM activates the pump in the sense that it is protected from oxidant-induced inhibition as a result of the reduced glutathionylation of C46.

**Fig. 5:**
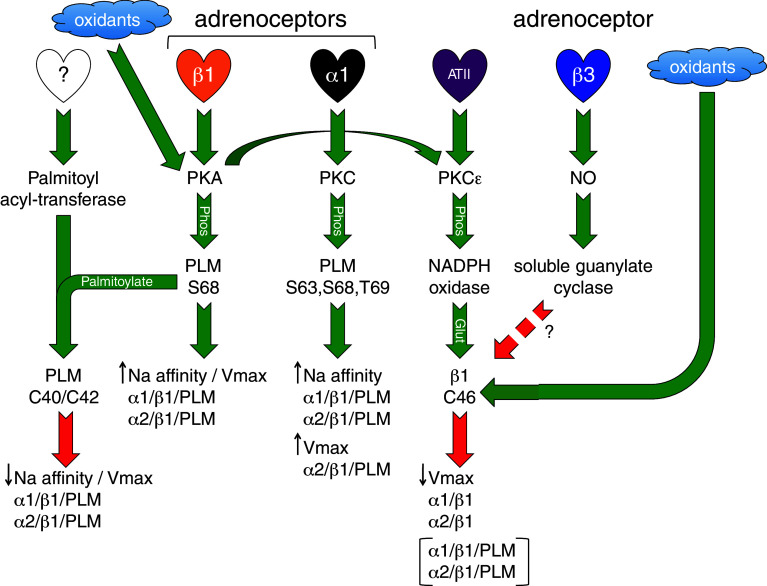
Summary of signaling pathways leading to sodium pump regulation in the heart. *Green arrows* indicate activation of the next step in the pathway, *red arrows* indicate inhibition. Full details are discussed in “Phospholemman the kinase target” to “Phospholemman: integrating multiple post-translational modifications?”

Again, the paradigm of PLM as a pump activator during redox signaling in cardiac muscle does not sit well with the consensus regarding the role of PLM in phosphoregulation of the pump. Multiple studies using numerous model systems agree that dephosphorylated PLM inhibits the pump (discussed above). Nor can redox and phosphoregulation by PLM be easily separated since it is now well established that oxidizing species activate PKA and PKG in ventricular muscle [165–167]. Indeed, hydrogen peroxide treatment of ventricular myocytes, which has been reported to inhibit the pump via β1 subunit glutathionylation [161], leads to substantial phosphorylation of PLM at S68 because it activates type 1 PKA (by promoting an inter-protein disulfide bond between its two regulatory subunits) [165]. Since phosphorylation of PLM at S68 promotes its palmitoylation [15], it is difficult to exactly predict the consequences for the pump. Palmitoylation and glutathionylation may compete for the same cysteine; the ability of this cysteine to receive either glutathione or palmitate will depend on whether it is already modified with the other. Hence, PLM may be a pump activator (through glutathionylation) or inhibitor (through palmitoylation) depending on the state of cysteine 42, which is determined by the phosphorylation status of PLM, adrenergic state of the tissue, and redox state of the cell. Figure 3 depicts some of the post-translational modifications of PLM discussed, and Fig. 5 summarizes our current knowledge of these pathways and how they may interact.

### Future directions

To date, remarkably few studies have investigated the effects of endogenous agonists of adrenoceptors on Na pump activity in ventricular muscle. Although a considerable amount of evidence points to the involvement of pathways linked to β1, β3 and α1 adrenoceptors, the additional activation of β2 adrenoceptors by adrenalin/noradrenalin must also be considered, as this generates a cAMP signal localized to the sarcolemma by caveolae (in which the Na pump resides, discussed below) [168, 169]. The consequences of the simultaneous activation of all the signaling pathways discussed above are hard to predict, and the balance between them will undoubtedly vary between health and disease. The multiprotein complexes that direct the different signaling events themselves remain largely unidentified. In addition, the use of endogenous agonists brings into play the endogenous uptake mechanisms for these agonists: the metabolism of adrenalin and noradrenalin taken up via uptake 2 in ventricular myocytes is through oxidative deamination by mitochondrial monoamine oxidase A, which generates hydrogen peroxide [170]. The oxidative burden imposed by this hydrogen peroxide (which will activate several of the pathways discussed above) can be significant enough to contribute to cardiac failure [170] and cell death during cardiac ischemia [171], and therefore warrants investigation in the context of the regulation of the cardiac Na pump by the adrenergic system.

## Direct regulation of the pump by small molecules

### Regulation by lipids

While perhaps not the subject of recent research, it is noteworthy that the cardiac Na pump is regulated by lipid species. Palmitoyl carnitine and lysophosphatidylcholine are potent pump inhibitors [172, 173], while long-chain fatty acyl CoA derivatives [174] and monoacylglycerols [175] stimulate the pump over concentration ranges it is likely to encounter in myocytes. These activators increase the pump’s ATP affinity, and it is suggested that they maintain Na pump activity against a backdrop of falling ATP during cardiac ischemia [174]. Sodium overload as a result of pump inhibition is a central feature of cardiac ischemia. In the ischemic heart, the pump is inhibited by the accumulation of a cytosolic substance whose production is inhibited by anti-oxidants [176], which is likely to be an oxidized lipid, but whose instability has precluded identification. The molecular identity of this inhibitor has recently been proposed to be oxidized glutathione [157].

While the stimulatory effect of long-chain acyl CoA on the pump is thought to be due to specific binding of the acyl-CoA to an intracellular domain of the pump, rather than non-specific effects on the bilayer [174], the mechanism by which lipids alter pump activity in the heart has not been rigorously investigated. It is now clear that the phospholipid environment in which the pump resides is one determinant of its activity. The cardiac pump is located in caveolae (discussed in “Caveolae and caveolins” below), which are rich in sphingolipids and cholesterol. In general, phospholipid interactions [177] and in particular cholesterol [178] are important to stabilize the pump, and PLM in particular of the FXYD family stabilizes the interaction between phosphatidylserine and the pump α subunit [164]. The functional relevance of this to the cardiac enzyme is unclear, since phosphatidylserine is without effect on pump activity [178]. That said, the stabilizing effect of PLM may be physiologically relevant: pump expression is reduced in the PLM KO heart [111, 179] and it has been suggested this may be due to the loss of the stabilizing effect of PLM making the pump more susceptible to degradation [118, 164]. It is proposed that phosphatidylserine stabilizes the pump through an interaction close to α subunit ninth transmembrane domain, which is where the FXYD protein also interacts [164]. PLM palmitoylation may therefore modify the relationship between the α subunit and phosphatidylserine, since the palmitate moieties present when PLM is palmitoylated at C40 and C42 must also be accommodated in the lipid bilayer. On the other hand although palmitoylation controls the turnover rate of PLM, it appears to do so independently of an effect on the degradation rate of the α subunit [15].

### Endogenous cardiotonic steroids

Although the cardiotonic effects of Na pump inhibitors such as digoxin and ouabain have been established for centuries, consensus on the finer molecular details of this phenomenon has been hard to reach. That the cardiotonic steroid binding site in the pump α subunit is physiologically important is demonstrated by its conservation across all vertebrates [180], although the sensitivity of the pump to ouabain does vary from species to species, with the rat and mouse particularly insensitive. The existence of endogenous ligands for this site was first proposed in the 1970s (reviewed in [180]), and anti-digoxin antibodies were successfully used to purify Na pump inhibitors from plasma in 1980 [181]. It is therefore perhaps remarkable that the chemical identity of this compound or group of compounds remains elusive [182]. Variously it has been identified as endogenous ouabain [183, 184], marinobufagenin [185], digoxin [186] and telocinobufagin [187]. Its physiological effect on blood pressure and heart rate is revealed by the use of the commercial anti-digoxin antibody Digibind, which reduces blood pressure and heart rate when administered to the brain [188], and reduces blood pressure when administered to many different models of hypertension (reviewed in [182]).

Positive inotropy following pump inhibition by cardiotonic steroids is as a result of reduced NCX activity increasing the calcium content of the SR. The pump isoform responsible for this phenomenon in the heart has been the subject of some debate. In rat ventricular myocytes, selective inhibition of α2-containing pumps (11 % of total pump) increases contractility without a global increase in intracellular sodium through the sodium-loading of a sub-sarcolemmal compartment functionally linked to NCX [24]. Through this functional coupling to NCX (co-ordinated by the linker protein ankyrin-B: see below), it has been proposed that the pump α2 subunit is a fundamental regulator of calcium handling and therefore contractility in the heart [189]. Experiments in transgenic animals in which the ouabain affinity of the α2 subunit has been reduced also clearly indicate that ouabain-induced inotropy requires inhibition of the α2-containing pumps [22, 190]. However when the ouabain sensitivity of the α1-containing pumps is enhanced by mutagenesis, these too are able to facilitate ouabain-induced increases in cardiac contractility in an NCX dependent manner [18]. Hence both isoforms of the pump mediate positive inotropy. The role of endogenous cardiotonic steroids in controlling cardiac inotropy is probably somewhat limited however, since baseline and β-adrenoceptor agonist-stimulated in vivo cardiac contractility is essentially identical between wild-type and all strains of ouabain affinity-modified mice [18, 190]. In animals with enhanced sensitivity of α1-containing pumps to ouabain, the hypertrophic response to pressure overload induced by aortic banding is elevated, implicating cardiotonic steroids in this pathological remodeling, but not in normal physiological cardiac function [191].

## Regulation by other associated proteins

### Caveolae and caveolins

Precise control of the subcellular location of the Na pump is essential for co-ordinated control of its activity. In cardiac muscle essentially all active pump is found in caveolae [192]. Caveolae are small flask-like invaginations of the cell membrane around 50–100 nm in diameter, found in almost all cells of the body. They represent a specialized form of lipid raft, an area of the cell membrane enriched in cholesterol and sphingolipids, characterized by the presence of the protein caveolin. The lipid environment, caveolin content and morphology of caveolae are central to their diverse functional roles, which include co-ordination of signal transduction, cholesterol homeostasis, and endocytosis. One of caveolae’s best-characterized roles is as a signalosome, a compartment that brings together components of signal transduction cascades (including receptors, effectors and targets) [193]. Within caveolae, the 20-residue scaffolding domain of caveolin (caveolin 3 in cardiac muscle) interacts with a complementary caveolin-binding domain in proteins, which enables oligomeric caveolin to act as a regulatory scaffold for macromolecular signaling complex formation [194]. Caveolae have been assigned a key role in regulation of signaling in the heart. For example, α1- and β2-adrenoceptors are found exclusively in caveolae-containing membrane fractions of the adult heart [195, 196], while β1-adrenoceptors are in both caveolae and the bulk sarcolemma [197]. Cardiac caveolae are also sites of enrichment of G proteins, including Gαs, Gi, and Gq [195, 198] (although see [196]). Effectors of adrenoceptors (including adenylyl cyclase V/VI, protein kinase A (RII), GRK2, phospholipase Cβ, PP2A and eNOS) are likewise concentrated in the cardiac caveolar domain [196–199], as are their downstream targets (for example, PLM). The distribution of receptors, effectors and their targets is key to the efficiency and fidelity of their coupling [168, 169, 196].

Apart from the Na pump, a considerable number of cardiac ion transporters are resident in cardiac caveolae: voltage-gated sodium channels [200], L-type calcium channels [199], voltage gated potassium channels [201], ATP-sensitive potassium channels [202], NCX1 [203] (although this has been challenged [204]), and the plasma membrane calcium ATPase (PMCA) [205]. Physical co-localization of ion transporters in the caveolar compartment may functionally link ion flow by providing a restricted diffusional space [206] and facilitates hormonal regulation of these transporters by placing them physically adjacent to signaling molecules. For example, regulation of a sub-population of L-type calcium channels by β2-adrenoceptors requires their colocalization in caveolae [199]. Furthermore, the presence of ion transporters in caveolae is likely to have functional relevance beyond signal transduction since the lipid composition of the bilayer in which an ion transporter resides is likely to influence its activity. Membrane cholesterol modulates many aspects of ion channel function: activity of the Na pump, for example, is regulated [207] and stabilized (discussed above [178]) by the cholesterol content of the membranes within which it resides.

The presence of the cardiac pump in caveolae is achieved through the presence of a caveolin binding motif [208]: either φ*XXXX*φ*XX*φ at the N terminus, or φ*X*φ*XXXX*φ at the C terminus, where φ represents an aromatic amino acid [209]. These motifs are highly conserved between isoforms and species. Although only ~30 % of cardiac α subunit is found in caveolin-enriched microdomains purified by sucrose gradient centrifugation from ventricular myocytes [210], essentially 100 % of the β subunit is in these microdomains [192]. Given the well-established requirement for the β subunit to form a functional pump, it is likely that non-caveolar α subunit represents pools from both biosynthetic and degradation pathways: the majority of pump activity (~75 %) is caveolar [192].

The relative functional concentration of pump isoforms in cardiac t-tubules [19, 24] must be reconciled with the finding that the majority of cardiac αβ is localized to buoyant caveolin-enriched membranes [192]. Whether caveolae are found in cardiomyocyte t-tubules has been the subject of some debate. Mature skeletal muscle t-tubules are reported to be largely free of caveolin 3 by some researchers [211], but not others [212]. However immunofluorescent (for example [199, 213]) and electron microscopy [214, 215] studies clearly indicate that both caveolin 3 and intact caveolae are found in the t-tubule system of ventricular muscle, albeit only in regions outside the dyad. Functionally, localization of the pump to cardiac caveolae is likely to be important to achieve colocalization with the signaling complexes that regulate it: PKA [196, 197], PKC isoforms (which migrate into caveolae upon activation [216]) and NADPH oxidase [84]. The local protein composition of pump-containing caveolae, which remains largely un-investigated, is clearly important both for acute pump regulation as well as in establishing local ion gradients and sub-sarcolemmal pools of sodium.

### Ankyrin-B

In addition to the well-characterized interaction between the cardiac Na pump and NCX1 [18, 189, 190], the pump is a member of a much larger multi-protein complex in cardiomyocyte t-tubules, which also includes the SR IP3-receptor, and is co-ordinated by the cytoskeletal linker protein ankyrin-B [217]. It has long been known that ankyrin-B binds the Na pump [218] at a conserved ALLK motif in the large third intracellular loop that contains the active site [219]. The role of this interaction is well understood for the localization of the pump to the basolateral membrane in polarized epithelia [220, 221], and its importance in cardiac muscle is now emerging. An ankyrin-B loss-of-function mutation (E1425G) is the basis of type 4 long QT syndrome as a result of disrupted co-ordination of the pump/NCX/IP3-receptor complex leading to calcium mishandling and arrhythmias [222]. The physical colocalization, and therefore functional coupling of these proteins is severely impaired in animals heterozygous for ankyrin-B knockout [217]. Therefore a second macromolecular complex exists in cardiac t-tubules, distinct from the classic ryanodine receptor/L-type calcium channel dyad. This complex is unique to cardiac muscle (ankyrin-B expression being ~10-fold lower in skeletal muscle [217]), and appears to be an adaption to optimize calcium handling by recruiting and functionally coupling the Na pump and NCX1 in cardiomyocyte t-tubules.

## Cardiac sodium pump as a therapeutic target in cardiovascular disease

A reduction of the transarcolemmal Na gradient in cardiac myocytes has been implicated in a variety of pathologies including ischemia/reperfusion [223, 224], hypertrophy and heart failure (HF) [123, 124, 225–227]. While a component of the elevation of Na in hypertrophy and HF *may* reflect an increase in sodium influx [52], there is a large body of evidence showing that Na pump function may also be compromised in cardiac hypertrophy [225–227] and failure [228].

In hypertrophy and HF many aspects of E–C coupling are clearly altered, however, the elevation in intracellular sodium may contribute to (1) the negative force-frequency relationship, (2) slowed relaxation (3) arrhythmias, and (4) impaired mitochondrial energetics [123, 229, 230]. In an elegant series of studies, O’Rourke and colleagues have recently shown that mitochondrial calcium plays a key role in linking ATP production to ATP demand (i.e., mechanical activity) [229–232]. Mitochondria take up calcium via a uniporter, and extrude it using a Na/Ca exchanger. Fast calcium transients in cardiac mitochondria match those in the myocyte cytosol [233]: as calcium rises in the cell, so does mitochondrial calcium and this activates Krebs Cycle dehydrogenases to increase NAD reduction to NADH, and therefore step-up ATP production [232]. This relationship, which crucially matches ATP supply to demand, is blocked when cytosolic sodium is elevated [233]: the rise in sodium activates Na/Ca exchange in the inner mitochondrial membrane and this keeps mitochondrial calcium low preventing ATP supply meeting demand—leaving the heart metabolically compromised. In myocytes from failing hearts in which cytosolic sodium is elevated, blockade of mitochondrial Na/Ca exchange is sufficient to restore mitochondrial function by enhancing mitochondrial calcium accumulation [229]. Not only might elevated cytosolic sodium contribute to the known metabolic insufficiency in hypertrophied hearts but Kohlhaas et al. [232] have shown that this increases mitochondrial free radical formation in failing hearts further exacerbating injury. Moreover, acute cardiac glycoside toxicity (assessed by increased intracellular sodium, reduced mitochondrial calcium and increased reactive oxygen species production in isolated myocytes) is ameliorated by inhibition of the mitochondrial Na/Ca exchanger with CGP-37157, while in vivo the positive inotropic effect of ouabain is actually enhanced by CGP-37157 [231].

This raises the interesting paradigm that strategies that *increase* myocyte sodium efflux, and therefore restore the sodium gradient, may be an effective means to restore mitochondrial ‘supply–demand’ matching in hypertrophy and failure. The positive effect on the Na pump of agents used in the treatment of cardiac hypertrophy and failure has been noted by others: ACE inhibitors, angiotensin receptor blockers, aldosterone antagonists, NO-donors and insulin are all known to stimulate the Na pump and, in clinical trials, these agents have all been shown to be protective [234]. Clearly this is in direct contradiction to the historic use of cardiotonic steroids as inotropes, and remains untested either in animal models or in the clinic. Digitalis, however, appears to provide symptomatic relief rather than a positive effect on long-term prognosis. In the largest trial of its kind, the Digitalis Investigation Group showed digoxin reduced hospitalization due to worsening HF symptoms but had no long-term effect on mortality [235]. Whether the functional benefit of pump stimulators to treat hypertrophy and failure (in terms of restoration of mitochondrial demand coupling) will outweigh the functional impediment (likely as a result of increased NCX activity unloading the SR) remains to be seen. Indeed, the ideal pharmacology would seem to be a combination of α1-pump activation *and* α2-pump inhibition to calcium-load the SR via NCX without imposing a significant sodium load [24, 189, 190]. Time will tell whether we can take advantage of our hard-won knowledge of the cardiac pump to achieve such a clinical application.

## Concluding remarks

In conclusion, all manner of regulatory pathways converge on the cardiac Na pump, and it sits exquisitely poised to respond to the changing demands of the cardiovascular system. To some extent a number of these regulatory pathways seem to cancel each other out, while others complement each other, and this raises questions such as ‘*why is it essential to fine*-*tune intracellular sodium so precisely?’* and ‘*which regulatory pathways are the most important?*’. It is undoubtedly not necessary to fine-tune intracellular sodium to the nearest micromolar, but it is perhaps not precision but *accuracy* that is important for the cardiac Na pump. That is, the set-point for intracellular sodium and Na pump activity may not need to be precisely clamped, but rather need to vary depending on prevailing physiological conditions (heart rate, sympathetic drive and so on). As for which are the most important regulatory pathways, the simplest answer may be that it depends on the state of the heart when it receives a particular signal whether that signal leads to Na pump activation or inhibition. Hence one can conceive of adrenoceptor activation leading to kinase-induced pump activation via PLM in the absence of redox stress, *and* oxidant-induced pump inhibition in a heart with reduced anti-oxidant reserve. That said, the most compelling information is perhaps that PLM knockout myocytes are more prone to calcium-overload induced arrhythmias when treated with a β adrenoceptor agonist [125]. This unequivocally highlights the protective role of PLM as a pump activator following adrenergic stimulation in the heart—although it does not address the relative contributions of pump activation via PLM phosphorylation versus pump disinhibition via β1 subunit deglutathionylation, which must perhaps wait for new transgenic models to be developed.

Another crucial factor in understanding the control of the cardiac pump is to appreciate the importance of local signaling networks and local ion gradients. This is not simply distinguishing t-tubular from surface sarcolemmal pumps, or α1 from α2 pumps, but drilling down into identifying subpopulations of pumps, the ion transporters with which they co-localize, the regulatory mechanisms that control them, their physiological significance, and measuring the sodium concentration ‘seen’ by each subpopulation. In other words our existing experimental tools must be improved so that we do not simply rely on whole cell pump currents and fluorescence measurements as indices of activity and substrate availability. Cardiac myocytes are complicated beasts: we are already accumulating evidence for local ion gradients and functional coupling of some but not all pumps to other sarcolemmal ion transporters. To continue to investigate global rather than local pump control is to ignore the reductionist approach that has served so well in the first 50 years of investigating this remarkable, ubiquitous enzyme complex.

### Electronic supplementary material

Below is the link to the electronic supplementary material.

Supplementary material 1 (DOC 213 kb)

## Abbreviations

CFP: Cyan fluorescent protein
E–C: Excitation–contraction
FRET: Fluorescence resonance energy transfer
PLB: Phospholamban
PLM: Phospholemman
PKA: Protein kinase A
PKC: Protein kinase C
NCX: Sodium-calcium exchanger
NO: Nitric oxide
SR: Sarcoplasmic reticulum
YFP: Yellow fluorescent protein
