# Supplementary material for: Regulation of the cardiac sodium pump
Source: Cell Mol Life Sci. 2012 Sep 7;70(8):1357–80. doi: 10.1007/s00018-012-1134-y (PMC3607738; doi:10.1007/s00018-012-1134-y)
Supplement: Supplementary file 1 — Supplementary material 1 (DOC 213 kb) [file 18_2012_1134_MOESM1_ESM.doc]

Data supplement: Sodium Pump alpha1 and alpha2 alignment

In the alignment below:

1. 85+ % conserved (where both a1 and a2 are the same, allowing only 1 outlier in both groups (both a1 and a2 – see residue 2 as example). Dark blue in heat map
2. Either no consensus for a1 and a2, or used if a1 and a2 cannot be discriminated significantly. Light blue in heat map
3. Conservative change (eg, D/E – see residue 8 for example), represented by ; in clustal. Also used even if there is a single outlier in one group (see residue 3 as example). Yellow in heat map
4. Moderate change (eg, V/A or P/A – see residue 5 as example). Represented by . in clustal. Orange in heap map
5. Major change (eg, Y/S – see residue Y as example). Also used even if a single outlier is present. If multiple outliers are present this is downgraded to 4. Red in heat map.

dog2 MGRGAGREYSPAATTAENGGGK--KKQKEKELDELKKEVAMDDHKLSLDELGRKYQVDLS 58

horse2 MGRGAGREYSPAATTAENGGGK--KKQKEKELDELKKEVAMDDHKLSLDELGRKYQVDLS 58

human2 MGRGAGREYSPAATTAENGGGK--KKQKEKELDELKKEVAMDDHKLSLDELGRKYQVDLS 58

mouse2 MGRGAGREYSPAATTAENGGGK--KKQKEKELDELKKEVAMDDHKLSLDELGRKYQVDLS 58

rabbit2 MGRGAGREYSPAATTAENGGGK--KKQKEKELDELKKEVAMDDHKLSLDELGRKYQVDLS 58

pig2 MGRGAGREYSPAATTAENGGGK--KKQKEKELDELKKEVAMDDHKLSLDELGRKYQVDLS 58

cow2 MGRGAGREYSPAATTAENGGGK--KKQKEKELDELKKEVAMDDHKLSLDELGRKYQVDLS 58

elephant2 -GRGAGREYSPAATTAENGGGK--KKQKEKELDELKKEVAMDDHKLSLDELGRKYQVDLS 57

pig1 MGKGVGRDKYEPAAVSEHGDKK--KAKKERDMDELKKEVSMDDHKLSLDELHRKYGTDLS 58

human1 MGKGVGRDKYEPAAVSEQGDKKGKKGKKDRDMDELKKEVSMDDHKLSLDELHRKYGTDLS 60

dog1 MGKGVGRDKYEPAAVSEHGDKK--KAKKERDMDELKKEVSMDDHKLSLDELHRKYGTDLS 58

cow1 MGKGVGRDKYEPAAVSEHGDKK--KAKKERDMDELKKEVSMDDHKLSLDELHRKYGTDLS 58

sheep1 MGKGVGRDKYEPAAVSEHGDKK--KAKKERDMDELKKEVSMDDHKLSLDELHRKYGTDLN 58

mouse1 MGKGVGRDKYEPAAVSEHGDKKGKKAKKERDMDELKKEVSMDDHKLSLDELHRKYGTDLS 60

rabbit1 MGKGVGRDKYEPAAVSEHGDKKGKKAKKERDMDELKKEVSMDDHKLSLDELHRKYGTDLS 60

Elephant1 MAFKVGRDKYEPAAVSEHGDKK--KAKKERDMDELKKEVTMDDHKLSLDELHRKYGTDLS 58

horse1 MGKGVGRDKYEPAAISEHGNKK--KAKKERDMDELKKEVSMDDHKLSLDELQRKYGTDLS 58

. .**: .*: :*:*. * * :*::::*******:*********** *** .**.

1131411355541453131451 153113331111111311111111111411154111

dog2 KGLTNQRAQDILARDGPNALTPPPTTPEWVKFCRQLFGGFSILLWIGAILCFLAYGIQAA 118

horse2 KGLTNQRAQDILARDGPNALTPPPTTPEWVKFCRQLFGGFSILLWIGAILCFLAYGIQAA 118

human2 KGLTNQRAQDVLARDGPNALTPPPTTPEWVKFCRQLFGGFSILLWIGAILCFLAYGIQAA 118

mouse2 KGLTNQRAQDILARDGPNALTPPPTTPEWVKFCRQLFGGFSILLWIGALLCFLAYGILAA 118

rabbit2 KGLTNQRAQDILARDGPNALTPPPTTPEWVKFCRQLFGGFSILLWIGAILCFLAFGIQAA 118

pig2 KGLTNQRAQDILARDGPNALTPPPTTPEWVKFCRQLFGGFSILLWIGAILCFLAYGIQAA 118

cow2 KGLTNQRAQDILARDGPNALTPPPTTPEWVKFCRQLFGGFSILLWIGAILCFLAFGIQAA 118

elephant2 KGLTNQRAQDILARDGPNALTPPPTTPEWVKFCRQLFGGFSILLWIGAILCFLAFGIQAA 117

pig1 RGLTPARAAEILARDGPNALTPPPTTPEWVKFCRQLFGGFSMLLWIGAILCFLAYGIQAA 118

human1 RGLTSARAAEILARDGPNALTPPPTTPEWIKFCRQLFGGFSMLLWIGAILCFLAYSIQAA 120

dog1 RGLTTARAAEILARDGPNALTPPPTTPEWVKFCRQLFGGFSMLLWIGAILCFLAYGIQAA 118

cow1 RGLTTARAAEILARDGPNALTPPPTTPEWVKFCRQLFGGFSMLLWIGAVLCFLAYGIQAA 118

sheep1 RGLTTARAAEILARDGPNALTPPPTTPEWVKFCRQLFGGFSMLLWIGAVLCFLAYGIQAA 118

mouse1 RGLTPARAAEILARDGPNALTPPPTTPEWVKFCRQLFGGFSMLLWIGAILCFLAYGIRSA 120

rabbit1 RGLTTARAAEILARDGPNALTPPPTTPEWVKFCRQLFGGFSMLLWIGAILCFLAYGILAA 120

Elephant1 RGLTTARAAEILARDGPNALTPPPTTPEWVKFCRQLFGGFSMLLWIGAILCFLAYGILAA 118

horse1 RGLTTARAAEILARDGPNALTPPPTTPEWVKFCRQLFGGFSMLLWIGAILCFLAYGIQAA 118

:*** ** ::******************:***********:******:*****:.* :*

311125115311111111111111111111111111111113111111111111211211

dog2 MEDEPSNDNLYLGVVLAAVVIVTGCFSYYQEAKSSKIMDSFKNMVPQQALVVREGEKMQI 178

horse2 MEDEPSNDNLYLGVVLAAVVIVTGCFSYYQEAKSSKIMDSFKNMVPQQALVVREGEKMQI 178

human2 MEDEPSNDNLYLGVVLAAVVIVTGCFSYYQEAKSSKIMDSFKNMVPQQALVIREGEKMQI 178

mouse2 MEDEPSNDNLYLGIVLAAVVIVTGCFSYYQEAKSSKIMDSFKNMVPQQALVIREGEKMQI 178

rabbit2 MEDEPSNDNLYLGVVLAAVVIVTGCFSYYQEAKSSKIMDSFKNMVPQQALVVREGEKMQI 178

pig2 MEDEPSNDNLYLGVVLAAVVIVTGCFSYYQEAKSSKIMDSFKNMVPQQALVVREGEKMQI 178

cow2 MEDEPSNDNLYLGVVLAAVVIVTGCFSYYQEAKSSKIMDSFKNMVPQQALVVREGEKMQI 178

elephant2 MEDEPSNDNLYLGVVLAAVVIVTGCFSYYQEAKSSKIMDSFKNMVPQQALVVREGEKIQI 177

pig1 TEEEPQNDNLYLGVVLSAVVIITGCFSYYQEAKSSKIMESFKNMVPQQALVIRNGEKMSI 178

human1 TEEEPQNDNLYLGVVLSAVVIITGCFSYYQEAKSSKIMESFKNMVPQQALVIRNGEKMSI 180

dog1 TEEEPQNDNLYLGVVLSAVVIITGCFSYYQEAKSSKIMESFKNMVPQQALVIRNGEKMSI 178

cow1 TEEEPQNDNLYLGVVLSAVVIITGCFSYYQEAKSSKIMESFKNMVPQQALVIRNGEKMSI 178

sheep1 TEEEPQNDNLYLGVVLSAVVIITGCFSYYQEAKSSKIMESFKNMVPQQALVIRNGEKMSI 178

mouse1 TEEEPPNDDLYLGVVLSAVVIITGCFSYYQEAKSSKIMESFKNMVPQQALVIRNGEKMSI 180

rabbit1 TEEDFDNDNLYLGVVLAAVVIITGCFSYYQEAKSSKIMESFKNMVPQQALVIRNGEKMSI 180

Elephant1 TEEELENDNLYLGVVLSAVVIITGCFSYYQEAKSSKIMESFKNMVPQQALVVRNGEKMSI 178

horse1 TEEEPQNDNLYLGVVLSAVVIITGCFSYYQEAKSSKIMESFKNMVPQQALVVRNGEKMSI 178

*:: **:****:**:****:****************:************:*:***:.*

513122111111111131111311111111111111113111111111111213111141

dog2 NAEEVVVGDLVEVKGGDRVPADLRIISSHGCKVDNSSLTGESEPQTRSPEFTHENPLETR 238

horse2 NAEEVVVGDLVEVKGGDRVPADLRIISSHGCKVDNSSLTGESEPQTRSPEFTHENPLETR 238

human2 NAEEVVVGDLVEVKGGDRVPADLRIISSHGCKVDNSSLTGESEPQTRSPEFTHENPLETR 238

mouse2 NAEEVVVGDLVEVKGGDRVPADLRIISSHGCKVDNSSLTGESEPQTRSPEFTHENPLETR 238

rabbit2 NAEEVVVGDLVEVKGGDRVPADLRIISSHGCKVDNSSLTGESEPQTRSPEFTHENPLETR 238

pig2 NAEEVVVGDLVEVKGGDRVPADLRIISSHGCKVDNSSLTGESEPQTRSPEFTHENPLETR 238

cow2 NAEEVVVGDLVEVKGGDRVPADLRIISSHGCKVDNSSLTGESEPQTRSPEFTHENPLETR 238

elephant2 NAEEVVVGDLVEVKGGDRVPADLRIISSHGCKVDNSSLTGESEPQTRSPEFTHENPLETR 237

pig1 NAEEVVVGDLVEVKGGDRIPADLRIISANGCKVDNSSLTGESEPQTRSPDFTNENPLETR 238

human1 NAEEVVVGDLVEVKGGDRIPADLRIISANGCKVDNSSLTGESEPQTRSPDFTNENPLETR 240

dog1 NAEEVVIGDLVEVKGGDRIPADLRIISANGCKVDNSSLTGESEPQTRSPDFTNENPLETR 238

cow1 NAEEVVVGDLVEVKGGDRIPADLRIISANGCKVDNSSLTGESEPQTRSPDFTNENPLETR 238

sheep1 NAEEVVVGDLVEVKGGDRIPADLRIISANGCKVDNSSLTGESEPQTRSPDFTNENPLETR 238

mouse1 NAEDVVVGDLVEVKGGDRIPADLRIISANGCKVDNSSLTGESEPQTRSPDFTNENPLETR 240

rabbit1 NAEDVVVGDLVEVKGGDRIPADLRIISANGCKVDNSSLTGESEPQTRSPDFTNENPLETR 240

Elephant1 NAEDVVVGDLVEVKGGDRIPADLRIISANGCKVDNSSLTGESEPQTRSPDFTNENPLETR 238

horse1 NAEEVVVGDLVEVKGGDRIPADLRIISANGCKVDNSSLTGESEPQTRSPDFTNENPLETR 238

***:**:***********:********::********************:**:*******

111111111111111111311111111331111111111111111111131131111111

dog2 NICFFSTNCVEGTARGIVIATGDRTVMGRIATLASGLEVGRTPIAMEIEHFIQLITGVAV 298

horse2 NICFFSTNCVEGTARGIVIATGDRTVMGRIATLASGLEVGRTPIAMEIEHFIQLITGVAV 298

human2 NICFFSTNCVEGTARGIVIATGDRTVMGRIATLASGLEVGRTPIAMEIEHFIQLITGVAV 298

mouse2 NICFFSTNCVEGTARGIVIATGDRTVMGRIATLASGLEVGQTPIAMEIEHFIQLITGVAV 298

rabbit2 NICFFSTNCVEGTARGIVIATGDRTVMGRIATLASGLEVGRTPIAMEIEHFIQLITGVAV 298

pig2 NICFFSTNCVEGTARGIVIATGDRTVMGRIATLASGLEVGRTPIAMEIEHFIQLITGVAV 298

cow2 NICFFSTNCVEGTARGIVIATGDRTVMGRIATLASGLEVGRTPIAMEIEHFIQLITGVAV 298

elephant2 NICFFSTNCVEGTARGIVIATGDRTVMGRIATLASGLEVGRTPIAMEIEHFIQLITGVAV 297

pig1 NIAFFSTNCVEGTARGIVVYTGDRTVMGRIATLASGLEGGQTPIAAEIEHFIHIITGVAV 298

human1 NIAFFSTNCVEGTARGIVVYTGDRTVMGRIATLASGLEGGQTPIAAEIEHFIHIITGVAV 300

dog1 NIAFFSTNCVKGTARGIVVYTGDRTVMGRIATLASGLEGGQTPIAAEIEHFIHIITGVAV 298

cow1 NIAFFSTNCVEGTARGIVVYTGDRTVMGRIATLASGLEGGQTPIAAEIEHFIHIITGVAV 298

sheep1 NIAFFSTNCVEGTARGIVVYTGDRTVMGRIATLASGLEGGQTPIAAEIEHFIHIITGVAV 298

mouse1 NIAFFSTNCVEGTARGIVVYTGDRTVMGRIATLASGLEGGQTPIAEEIEHFIHLITGVAV 300

rabbit1 NIAFFSTNCVEGTARGIVIYTGDRTVMGRIATLASGLEGGQTPIAAEIEHFIHIITGVAV 300

Elephant1 NIAFFSTNCVEGTARGIVIYTGDRTVMGRIATLASGLEGGQTPIAAEIEHFIHIITGVAV 298

horse1 NIAFFSTNCVEGTARGIVVYTGDRTVMGRIATLASGLEGGQTPIAAEIEHFIHIITGVAV 298

**.*******:*******: ****************** *:**** ******::******

114111111111111111151111111111111111115131111511111133111111

dog2 FLGVSFFVLSLILGYSWLEAVIFLIGIIVANVPEGLLATVTVCLTLTAKRMARKNCLVKN 358

horse2 FLGVSFFVLSLILGYSWLEAVIFLIGIIVANVPEGLLATVTVCLTLTAKRMARKNCLVKN 358

human2 FLGVSFFVLSLILGYSWLEAVIFLIGIIVANVPEGLLATVTVCLTLTAKRMARKNCLVKN 358

mouse2 FLGVSFFVLSLILGYSWLEAVIFLIGIIVANVPEGLLATVTVCLTLTAKRMARKNCLVKN 358

rabbit2 FLGVSFFVLSLILGYSWLEAVIFLIGIIVANVPEGLLATVTVCLTLTAKRMARKNCLVKN 358

pig2 FLGVSFFVLSLILGYSWLEAVIFLIGIIVANVPEGLLATVTVCLTLTAKRMARKNCLVKN 358

cow2 FLGVSFFVLSLILGYSWLEAVIFLIGIIVANVPEGLLATVTVCLTLTAKRMARKNCLVKN 358

elephant2 FLGVSFFVLSLILGYSWLEAVIFLIGIIVANVPEGLLATVTVCLTLTAKRMARKNCLVKN 357

pig1 FLGVSFFILSLILEYTWLEAVIFLIGIIVANVPEGLLATVTVCLTLTAKRMARKNCLVKN 358

human1 FLGVSFFILSLILEYTWLEAVIFLIGIIVANVPEGLLATVTVCLTLTAKRMARKNCLVKN 360

dog1 FLGVSFFILSLILEYTWLEAVIFLIGIIVANVPEGLLATVTVCLTLTAKRMARKNCLVKN 358

cow1 FLGVSFFILSLILEYTWLEAVIFLIGIIVANVPEGLLATVTVCLTLTAKRMARKNCLVKN 358

sheep1 FLGVSFFILSLILEYTWLEAVIFLIGIIVANVPEGLLATVTVCLTLTAKRMARKNCLVKN 358

mouse1 FLGVSFFILSLILEYTWLEAVIFLIGIIVANVPEGLLATVTVCLTLTAKRMARKNCLVKN 360

rabbit1 FLGVSFFILSLILEYTWLEAVIFLIGIIVANVPEGLLATVTVCLTLTAKRMARKNCLVKN 360

Elephant1 FLGVSFFILSLILEYTWLEAVIFLIGIIVANVPEGLLATVTVCLTLTAKRMARKNCLVKN 358

horse1 FLGVTFFILSLILEYTWLEAVIFLIGIIVANVPEGLLATVTVCLTLTAKRMARKNCLVKN 358

****:**:***** *:********************************************

111111131111151311111111111111111111111111111111111111111111

dog2 LEAVETLGSTSTICSDKTGTLTQNRMTVAHMWFDNQIHEADTTEDQSGATFDKRSPTWTA 418

horse2 LEAVETLGSTSTICSDKTGTLTQNRMTVAHMWFDNQIHEADTTEDQSGATFDKRSPTWTA 418

human2 LEAVETLGSTSTICSDKTGTLTQNRMTVAHMWFDNQIHEADTTEDQSGATFDKRSPTWTA 418

mouse2 LEAVETLGSTSTICSDKTGTLTQNRMTVAHMWFDNQIHEADTTEDQSGATFDKRSPTWTA 418

rabbit2 LEAVETLGSTSTICSDKTGTLTQNRMTVAHMWFDNQIHEADTTEDQSGATFDKRSPTWTA 418

pig2 LEAVETLGSTSTICSDKTGTLTQNRMTVAHMWFDNQIHEADTTEDQSGATFDKRSPTWTA 418

cow2 LEAVETLGSTSTICSDKTGTLTQNRMTVAHMWFDNQIHEADTTEDQSGATFDKRSPTWTA 418

elephant2 LEAVETLGSTSTICSDKTGTLTQNRMTVAHMWFDNQIHEADTTEDQSGATFDKRSPTWTA 417

pig1 LEAVETLGSTSTICSDKTGTLTQNRMTVAHMWSDNQIHEADTTENQSGVSFDKTSATWLA 418

human1 LEAVETLGSTSTICSDKTGTLTQNRMTVAHMWFDNQIHEADTTENQSGVSFDKTSATWLA 420

dog1 LEAVETLGSTSTICSDKTGTLTQNRMTVAHMWFDNQIHEADTTENQSGVSFDKSSATWLA 418

cow1 LEAVETLGSTSTICSDKTGTLTQNRMTVAHMWFDNQIHEADTTENQSGVSFDKTSATWLA 418

sheep1 LEAVETLGSTSTICSDKTGTLTQNRMTVAHMWFDNQIHEADTTENQSGVSFDKTSATWLA 418

mouse1 LEAVETLGSTSTICSDKTGTLTQNRMTVAHMWFDNQIHEADTTENQSGVSFDKTSATWFA 420

rabbit1 LEAVETLGSTSTICSDKTGTLTQNRMTVAHMWFDNQIHEADTTENQSGVSFDKTSATWLA 420

Elephant1 LEAVETLGSTSTICSDKTGTLTQNRMTVAHMWFDNQIHEADTTENQSGVSFDKSSITWLA 418

horse1 LEAVETLGSTSTICSDKTGTLTQNRMTVAHMWFDNQIHEADTTENQSGVSFDKTSATWLS 418

******************************** ***********:***.:*** * ** :

111111111111111111111111111111111111111111113111431115151151

dog2 LSRIAGLCNRAVFKAGQENISVSKRD-TAGDASESALLKCIELSCGSVRKMRDRNPKVAE 477

horse2 LSRIAGLCNRAVFKAGQENISVSKRDTTRGMPPESALLKCIELSCGSCGRXGDRNPKVAE 478

human2 LSRIAGLCNRAVFKAGQENISVSKRD-TAGDASESALLKCIELSCGSVRKMRDRNPKVAE 477

mouse2 LSRIAGLCNRAVFKAGQENISVSKRD-TAGDASESALLKCIELSCGSVRKMRDRNPKVAE 477

rabbit2 LSRIAGLCNRAVFKAGQENISVSKRD-TAGDASESALLKCIELSCGSVRKMRDRNPKVAE 477

pig2 LSRIAGLCNRAVFKAGQENISVSKRD-TAGDASESALLKCIELSCGSVRKMRDRNPKVAE 477

cow2 LSRIAGLCNRAVFKAGQENISVSKRD-TAGDASESALLKCIELSCGSVRKMRDRNPKVAE 477

elephant2 LSRIAGLCNRAVFKAGQENISVSKRD-TAGDASESALLKCIELSCGSVKKMRERNPKVAE 476

pig1 LSRIAGLCNRAVFQANQENLPILKRA-VAGDASESALLKCIELCCGSVKEMRERYTKIVE 477

human1 LSRIAGLCNRAVFQANQENLPILKRA-VAGDASESALLKCIELCCGSVKEMRERYAKIVE 479

dog1 LSRIAGLCNRAVFQANQENLPILKRA-VAGDASESALLKCIELCCGSVKEMRDRYAKIVE 477

cow1 LSRIAGLCNRAVFQANQDNLPILKRA-VAGDASESALLKCIEVCCGSVKEMRERYTKIVE 477

sheep1 LSRIAGLCNRAVFQANQDNLPILKRA-VAGDASESALLKCIEVCCGSVKEMRERYAKIVE 477

mouse1 LSRIAGLCNRAVFQANQENLPILKRA-VAGDASESALLKCIEVCCGSVMEMREKYSKIVE 479

rabbit1 LSRIAGLCNRAVFQANQENLPILKRA-VAGDASESALLKCIELCCGSVKEMRERYTKIVE 479

Elephant1 LSRIAGLCNRAVFQANQENIPILKRA-VAGDASESALLKCIELCCGSVKEMREQYTKIVE 477

horse1 LSRIAGLCNRAVFQANQENIPILKRA-VAGDASESALLKCIELCCGSVKEMRDRYPKIVE 477

*************:*.*:*:.: ** . * ..*********:.*** . :: .*:.*

11111111111113141112435115 411111111111111231111241121521341

dog2 IPFNSTNKYQLSIHEREDS-PQSHVLVMKGAPERILDRCSTILVQGKEIPLDKEMQDAFQ 536

horse2 IPFNSTNKYQLSIHEREDS-PQSHVLVMKGAPERILDRCSTILVQGKEIPLDKEMQDAFQ 537

human2 IPFNSTNKYQLSIHEREDS-PQSHVLVMKGAPERILDRCSTILVQGKEIPLDKEMQDAFQ 536

mouse2 IPFNSTNKYQLSIHEREDS-PQSHVLVMKGAPERILDRCSTILVQGKEIPLDKEMQDAFQ 536

rabbit2 IPFNSTNKYQLSIHEREDS-PQSHVLVMKGAPERILDRCSTILVQGKEIPLDKEMQDAFQ 536

pig2 IPFNSTNKYQLSIHEREDN-PQSHVLVMKGAPERILDRCSSILVQGKEIPLDKEMQDAFQ 536

cow2 IPFNSTNKYQLSIHEREDS-PQSHVLVMKGAPERILDRCSSILVQGKEIPLDKEMQDAFQ 536

elephant2 IPFNSTNKYQLSIHEREDS-PQSHVLVMKGAPERILDRCSTILVQGKEIPLDKEMQDAFQ 535

pig1 IPFNSTNKYQLSIHKNPNTAEPRHLLVMKGAPERILDRCSSILIHGKEQPLDEELKDAFQ 537

human1 IPFNSTNKYQLSIHKNPNTSEPQHLLVMKGAPERILDRCSSILLHGKEQPLDEELKDAFQ 539

dog1 IPFNSTNKYQLSIHKNPNTSEPRHLLVMKGAPERILDRCSSILLHGKEQPLDEELKDALQ 537

cow1 IPFNSTNKYQLSIHKNANAGEPRHLLVMKGAPERILDRCSSILIHGKEQPLDEELKDAFQ 537

sheep1 IPFNSTNKYQLSIHKNANAGEPRHLLVMKGAPERILDRCSSILIHGKEQPLDEELKDAFQ 537

mouse1 IPFNSTNKYQLSIHKNPNASEPKHLLVMKGAPERILDRCSSILLHGKEQPLDEELKDAFQ 539

rabbit1 IPFNSTNKYQLSIHKNLNANEPRHLLVMKGAPERILDRCSSILLHGKEQPLDEELKDAFQ 539

Elephant1 IPFNSTNKYQLSIHKNPNTSEPRHLLVMKGAPERILDRCSSILLHGKEQPLDEELKDAFQ 537

horse1 IPFNSTNKYQLSIHKNPNTSEPQHLLVMKGAPERILDRCSSILLNGKEQPLDEELKDAFQ 537

**************:. : *:***************:**::*** ***:*::**:*

111111111111113453255551311111111111111131133111511131331111

dog2 NAYMELGGLGERVLGFCQLNLPSGKFPRGFRFDTDELNFPTEKLCFVGLMSMIDPPRAAV 596

horse2 NAYMELGGLGERVLGFCQLNLPSGKFPRGFKFDTDELNFPTEKLCFVGLMSMIDPPRAAV 597

human2 NAYMELGGLGERVLGFCQLNLPSGKFPRGFKFDTDELNFPTEKLCFVGLMSMIDPPRAAV 596

mouse2 NAYMELGGLGERVLGFCQLNLPSGKFPRGFKFDTDELNFPTEKLCFVGLMSMIDPPRAAV 596

rabbit2 NAYMELGGLGERVLGFCHLNLPSGKFPRGFKFDTDELNFPTEKLCFVGLMSMIDPPRAAV 596

pig2 NAYLELGGLGERVLGFCQLNLPSGKFPRGFKFDTDELNFPTEKLCFVGLMSMIDPPRAAV 596

cow2 NAYLELGGLGERVLGFCQLNLPSAKFPRGFKFDTDELNFPTEKLCFVGLMSMIDPPRAAV 596

elephant2 NAYMELGGLGERVLGFCHLNLPSGKFPRGFKFDTDELNFPTEKLCFVGLMSMIDPPRAAV 595

pig1 NAYLELGGLGERVLGFCHLFLPDEQFPEGFQFDTDDVNFPLDNLCFVGLISMIDPPRAAV 597

human1 NAYLELGGLGERVLGFCHLFLPDEQFPEGFQFDTDDVNFPIDNLCFVGLISMIDPPRAAV 599

dog1 NAYLELGGLGERVLGFRHLFLPDEQFPEGFQFDTDDVNFPVENLCFVGFISMIGPPRAAV 597

cow1 NAYLELGGLGERVLGFCHLLLPDEQFPEGFQFDTDDVNFPVDNLCFVGLISMIDPPRAAV 597

sheep1 NAYLELGGLGERVLGFCHLMLPDEQFPEGFQFDTDDVNFPVDNLCFVGLISMIDPPRAAV 597

mouse1 NAYLELGGLGERVLGFCHLLLPDEQFPEGFQFDTDDVNFPVDNLCFVGLISMIDPPRAAV 599

rabbit1 NAYLELGGLGERVLGFCHLLLPDEQFPEGFQFDTDEVNFPVDNLCFIGLISMIDPPRAAV 599

Elephant1 NAYLELGGLGERVLGFCHLSLPDEQFPEGFQFDTDDVNFPVDNLCFVGLISMIDPPRAAV 597

horse1 NAYLELGGLGERVLGFCHLFLPDEQFPEGFQFDTDDVNFPLENLCFVGLISMIDPPRAAV 597

***:************ :* **. :**.**:****::*** ::***:*::***.******

1112111111111111131**3**1145311411311113311152311111131111111111

dog2 PDAVGKCRSAGIKVIMVTGDHPITAKAIAKGVGIISEGNETVEDIAARLNIPVSQVNPRE 656

horse2 PDAVGKCRSAGIKVIMVTGDHPITAKAIAKGVGIISEGNETVEDIAARLNIPVSQVNPRE 657

human2 PDAVGKCRSAGIKVIMVTGDHPITAKAIAKGVGIISEGNETVEDIAARLNIPMSQVNPRE 656

mouse2 PDAVGKCRSAGIKVIMVTGDHPITAKAIAKGVGIISEGNETVEDIAARLNIPVSQVNPRE 656

rabbit2 PDAVGKCRSAGIKVIMVTGDHPITAKAIAKGVGIISEGNETVEDIAARLNIPVSQVNPRE 656

pig2 PDAVGKCRSAGIKVIMVTGDHPITAKAIAKGVGIISEGNETVEDIAARLNIPVSQVNPRE 656

cow2 PDAVGKCRSAGIKVIMVTGDHPITAKAIAKGVGIISEGNETVEDIAARLNIPVSQVNPRE 656

elephant2 PDAVGKCRSAGIKVIMVTGDHPITAKAIAKGVGIISEGNETVEDIAARLNIPVTQVNPRE 655

pig1 PDAVGKCRSAGIKVIMVTGDHPITAKAIAKGVGIISEGNETVEDIAARLNIPVSQVNPRD 657

human1 PDAVGKCRSAGIKVIMVTGDHPITAKAIAKGVGIISEGNETVEDIAARLNIPVSQVNPRD 659

dog1 PDAVGKCRGAGIKVIMVTGDHPITAKAIAKGAGIISEGNETVEDIAARLNIPVRQVNPRD 657

cow1 PDAVGKCRSAGIKVIMVTGDHPITAKAIAKGVGIISEGNETVEDIAARLNIPVSQVNPRD 657

sheep1 PDAVGKCRSAGIKVIMVTGDHPITAKAIAKGVGIISEGNETVEDIAARLNIPVSQVNPRD 657

mouse1 PDAVGKCRSAGIKVIMVTGDHPITAKAIAKGVGIISEGNETVEDIAARLNIPVNQVNPRD 659

rabbit1 PDAVGKCRSAGIKVIMVTGDHPITAKAIAKGVGIISEGNETVEDIAARLNIPVSQVNPRD 659

Elephant1 PDAVGKCRSAGIKVIMVTGDHPITAKAIAKGVGIISEGNETVEDIAARLNIPVSQVNPRD 657

horse1 PDAVGKCRSAGIKVIMVTGDHPITAKAIAKGVGIISEGNETVEDIAARLNIPVSQVNPRD 657

********.**********************.********************: *****:

111111111111111111111111111111111111111111111111111111111113

dog2 AKACVVHGSDLKDMTSEQLDEILKNHTEIVFARTSPQQKLIIVEGCQRQGAIVAVTGDGV 716

horse2 AKACVVHGSDLKDMTPEQLDEILKNHTEIVFARTSPQQKLIIVEGCQRQGAIVAVTGDGV 717

human2 AKACVVHGSDLKDMTSEQLDEILKNHTEIVFARTSPQQKLIIVEGCQRQGAIVAVTGDGV 716

mouse2 AKACVVHGSDLKDMTSEQLDEILRDHTEIVFARTSPQQKLIIVEGCQRQGAIVAVTGDGV 716

rabbit2 AKACVVHGSDLKDMTSEQLDEILRNHTEIVFARTSPQQKLIIVEGCQRQGAIVAVTGDGV 716

pig2 AKACVVHGSDLKDMTSEQLDEILKNHTEIVFARTSPQQKLIIVEGCQRQGAIVAVTGDGV 716

cow2 AKACVVHGSDLKDMTSEQLDEILKNHTEIVFARTSPQQKLIIVEGCQRQGAIVAVTGDGV 716

elephant2 AKACVVHGSDLKDMTAEQLDEILKNHTEIVFARTSPQQKLIIVEGCQRQGAIVAVTGDGV 715

pig1 AKACVVHGSDLKDMTSEQLDDILKYHTEIVFARTSPQQKLIIVEGCQRQGAIVAVTGDGV 717

human1 AKACVVHGSDLKDMTSEQLDDILKYHTEIVFARTSPQQKLIIVEGCQRQGAIVAVTGDGV 719

dog1 AKACVVHGSDLKDMTSEQLDGILKYHTEIVFARTSPQQKLIIVEGCQRQGAIVAVTGDGV 717

cow1 ARACVVHGSDLKDMTPEQLDDILKYHTEIVFARTSPQQKLIIVEGCQRQGAIVAVTGDGV 717

sheep1 ARACVVHGSDLKDMTPEQLDDILKYHTEIVFARTSPQQKLIIVEGCQRQGAIVAVTGDGV 717

mouse1 AKACVVHGSDLKDMTSEELDDILRYHTEIVFARTSPQQKLIIVEGCQRQGAIVAVTGDGV 719

rabbit1 AKACVVHGSDLKDMTSEQLDDILKYHTEIVFARTSPQQKLIIVEGCQRQGAIVAVTGDGV 719

Elephant1 AKACVVHGSDLKDMTPEQLDDILKYHTEIVFARTSPQQKLIIVEGCQRQGAIVAVTGDGV 717

horse1 AKACVVHGSDLKDMTPEQLDDILRHHTEIVFARTSPQQKLIIVEGCQRQGAIVAVTGDGV 717

*:*************.*:** **: ***********************************

111111111111111211113112211111111111111111111111111111111111

dog2 NDSPALKKADIGIAMGISGSDVSKQAADMILLDDNFASIVTGVEEGRLIFDNLKKSIAYT 776

horse2 NDSPALKKADIGIAMGISGSDVSKQAADMILLDDNFASIVTGVEEGRLIFDNLKKSIAYT 777

human2 NDSPALKKADIGIAMGISGSDVSKQAADMILLDDNFASIVTGVEEGRLIFDNLKKSIAYT 776

mouse2 NDSPALKKADIGIAMGISGSDVSKQAADMILLDDNFASIVTGVEEGRLIFDNLKKSIAYT 776

rabbit2 NDSPALKKADIGIAMGISGSDVSKQAADMILLDDNFASIVTGVEEGRLIFDNLKKSIAYT 776

pig2 NDSPALKKADIGIAMGIAGSDVSKQAADMILLDDNFASIVTGVEEGRLIFDNLKKSIAYT 776

cow2 NDSPALKKADIGIAMGIAGSDVSKQAADMILLDDNFASIVTGVEEGRLIFDNLKKSIAYT 776

elephant2 NDSPALKKADIGIAMGISGSDVSKQAADMILLDDNFASIVTGVEEGRLIFDNLKKSIAYT 775

pig1 NDSPASKKADIGVAMGIAGSDVSKQAADMILLDDNFASIVTGVEEGRLIFDNLKKSIAYT 777

human1 NDSPALKKADIGVAMGIAGSDVSKQAADMILLDDNFASIVTGVEEGRLIFDNLKKSIAYT 779

dog1 NDSPALKKADIGVAMGIVGSDASKQAADMILLDDNFASIVTGVEEGRLIFDNLKKSIAYT 777

cow1 NDSPALKKADIGVAMGIAGSDVSKQAADMILLDDNFASIVTGVEEGRLIFDNLKKSIAYT 777

sheep1 NDSPALKKADIGVAMGIAGSDVSKQAADMILLDDNFASIVTGVEEGRLIFDNLKKSIAYT 777

mouse1 NDSPALKKADIGVAMGIVGSDVSKQAADMILLDDNFASIVTGVEEGRLIFDNLKKSIAYT 779

rabbit1 NDSPALKKADIGVAMGIAGSDVSKQAADMILLDDNFASIVTGVEEGRLIFDNLKKSIAYT 779

Elephant1 NDSPALKKADIGVAMGIAGSDVSKQAADMILLDDNFASIVTGVEEGRLIFDNLKKSIAYT 777

horse1 NDSPALKKADIGVAMGIAGSDVSKQAADMILLDDNFASIVTGVEEGRLIFDNLKKSIAYT 777

***** ******:**** ***.**************************************

111111111111311112111111111111111111111111111111111111111111

dog2 LTSNIPEITPFLLFIIANIPLPLGTVTILCIDLGTDMVPAISLAYEAAESDIMKRQPRNP 836

horse2 LTSNIPEITPFLLFIIANIPLPLGTVTILCIDLGTDMVPAISLAYEAAESDIMKRQPRNS 837

human2 LTSNIPEITPFLLFIIANIPLPLGTVTILCIDLGTDMVPAISLAYEAAESDIMKRQPRNS 836

mouse2 LTSNIPEITPFLLFIIANIPLPLGTVTILCIDLGTDMVPAISLAYEAAESDIMKRQPRNS 836

rabbit2 LTSNIPEITPFLLFIIANIPLPLGTVTILCIDLGTDMVPAISLAYEAAESDIIDAAPRFL 836

pig2 LTSNIPEITPFLLFIIANIPLPLGTVTILCIDLGTDMVPAISLAYEAAESDIMKRQPRNP 836

cow2 LTSNIPEITPFLLFIIANIPLPLGTVTILCIDLGTDMVPAISLAYEAAESDIMKRQPRNP 836

elephant2 LTSNIPEITPFLMFIIVNIPLPLGTVTILCIDLGTDMVPAISLAYEAAESDIMKRQPRNP 835

pig1 LTSNIPEITPFLIFIIANIPLPLGTVTILCIDLGTDMVPAISLAYEQAESDIMKRQPRNP 837

human1 LTSNIPEITPFLIFIIANIPLPLGTVTILCIDLGTDMVPAISLAYEQAESDIMKRQPRNP 839

dog1 LTSNIPEITPFLIFIIANIPLPLGTVTILCIDLGTDMVPAISLAYEQAESDIMKRQPRNP 837

cow1 LTSNIPEITPFLIFIIANIPLPLGTVTILCIDLGTDMVPAISLAYEQAESDIMKRQPRNP 837

sheep1 LTSNIPEITPFLIFIIANIPLPLGTVTILCIDLGTDMVPAISLAYEQAESDIMKRQPRNP 837

mouse1 LTSNIPEITPFLIFIIANIPLPLGTVTILCIDLGTDMVPAISLAYEQAESDIMKRQPRNP 839

rabbit1 LTSNIPEITPFLIFIIANIPLPLGTVTILCIDLGTDMVPAISLAYEQAESDIMKRQPRNP 839

Elephant1 LTSNIPEITPFLIFIIANIPLPLGTVTILCIDLGTDMVPAISLAYEQAESDIMKRQPRNP 837

horse1 LTSNIPEITPFLIFIIANIPLPLGTVTILCIDLGTDMVPAISLAYEQAESDIMKRQPRNP 837

************:***.***************************** *****:. **

111111111111311111111111111111111111111111111151111111111112

dog2 QTDKLVNERLISMAYGQIGMIQALGGFFTYFVILAENGFLPSRLLGIRLDWDDRSMNDLE 896

horse2 QTDKLVNERLISMAYGQIGMIQALGGFFTYFVILAENGFLPSRLLGIRLDWDDRSMNDLE 897

human2 QTDKLVNERLISMAYGQIGMIQALGGFFTYFVILAENGFLPSRLLGIRLDWDDRTMNDLE 896

mouse2 QTDKLVNERLISMAYGQIGMIQALGGFFTYFVILAENGFLPSRLLGIRLDWDDRTTNDLE 896

rabbit2 RRDKLVNERLISMAYGQIGMIQALGGFFTYFVILAENGFLPSRLLGIRLDWDDRTMNDLE 896

pig2 QTDKLVNERLISMAYGQIGMIQALGGFFTYFVILAENGFLPSRLLGIRLDWDDRSMNDLE 896

cow2 QTDKLVNERLISMAYGQIGMIQALGGFFTYFVILAENGFLPSRLLGIRLDWDDRSMNDLE 896

elephant2 QTDKLVNERLISMAYGQIGMIQALGGFFTYFVILAENGFLPSRLLGIRLDWDDRSRNDLE 895

pig1 KTDKLVNEQLISMAYGQIGMIQALGGFFTYFVILAENGFLPIHLLGLRVNWDDRWINDVE 897

human1 KTDKLVNERLISMAYGQIGMIQALGGFFTYFVILAENGFLPIHLLGLRVDWDDRWINDVE 899

dog1 KTDKLVNERLISMAYGQIGMIQALGGFFTYFVILAENGFLPTHLLGLRVDWDDRWINDVE 897

cow1 QTDKLVNERLISMAYGQIGMIQALGGFFTYFVIMAENGFLPNHLLGIRVTWDDRWINDVE 897

sheep1 QTDKLVNERLISMAYGQIGMIQALGGFFTYFVIMAENGFLPNHLLGIRVTWDDRWINDVE 897

mouse1 KTDKLVNERLISMAYGQIGMIQALGGFFTYFVILAENGFLPFHLLGIRETWDDRWVNDVE 899

rabbit1 KTDKLVNERLISMAYGQIGMIQALGGFFTYFVILAENGFLPFHLLGIRVDWDDRWINDVE 899

Elephant1 QTDKLVNERLISMAYGQIGMIQALGGFFTYFVIMAENGFLPIDLLGLRVDWDDRWINDVE 897

horse1 QTDKLVNERLISMAYGQIGMIQALGGFFTYFVILAENGFLPIHLLGLRVDWDDRWVNDVE 897

: ******:************************:******* ***:* **** **:*

211111111111111111111111111111111211111112**3**11121321111521131

dog2 DSYGQEWTYEQRKVVEFTCHTAFFASIVVVQWADLIICKTRRNSVFQQGMKNKILIFGLL 956

horse2 DSYGQEWTYEQRKVVEFTCHTAFFASIVVVQWADLIICKTRRNSVFQQGMKNKILIFGLL 957

human2 DSYGQEWTYEQRKVVEFTCHTAFFASIVVVQWADLIICKTRRNSVFQQGMKNKILIFGLL 956

mouse2 DSYGQEWTYEQRKVVEFTCHTAFFASIVVVQWADLIICKTRRNSVFQQGMKNKILIFGLL 956

rabbit2 DSYGQEWTYEQRKVVEFTCHTAFFASIVVVQWADLIICKTRRNSVFQQGMKNKILIFGLL 956

pig2 DSYGQEWTYEQRKVVEFTCHTAFFASIVVVQWADLIICKTRRNSVFQQGMKNKILIFGLL 956

cow2 DSYGQEWTYEQRKVVEFTCHTAFFASIVVVQWADLIICKTRRNSVFQQGMKNKILIFGLL 956

elephant2 DSYGQEWTYEQRKVVEFTCHTAFFASIVVVQWADLIICKTRRNSVFQQGMKNKILVFGLL 955

pig1 DSYGQQWTYEQRKIVEFTCHTPFFVTIVVVQWADLVICKTRRNSVFQQGMKNKILIFGLF 957

human1 DSYGQQWTYEQRKIVEFTCHTAFFVSIVVVQWADLVICKTRRNSVFQQGMKNKILIFGLF 959

dog1 DSYGQQWTYEQRKIVEFTCHTAFFVSIVVVQWADLVICKTRRNSVFQQGMKNKILIFGLF 957

cow1 DSYGQQWTYEQRKIVEFTCHTAFFVSIVVVQWADLVICKTRRNSVFQQGMKNKILIFGLF 957

sheep1 DSYGQQWTYEQRKIVEFTCHTAFFVSIVVVQWADLVICKTRRNSVFQQGMKNKILIFGLF 957

mouse1 DSYGQQWTYEQRKIVEFTCHTAFFVSIVVVQWADLVICKTRRNSVFQQGMKNKILIFGLF 959

rabbit1 DSYGQQWTYEQRKIVEFTCHTAFFVSIVVVQWADLVICKTRRNSVFQQGMKNKILIFGLF 959

Elephant1 DSYGQQWTYEQRKIVEFTCHTAFFVSIVVVQWADLVICKTRRNSVFQQGMKNKILIFGLF 957

horse1 DSYGQQWTYEQRKIVEFTCHTAFFVSIVVVQWADLVICKTRRNSVFQQGMKNKILIFGLF 957

*****:*******:*******.**.:*********:*******************:***:

111113111111131111111111411111111113111111111111111111111113

dog2 EETALAAFLSYCPGMGVALRMYPLKVTWWFCAFPYSLLIFIYDEVRKLILRRYPGGWVEK 1016

horse2 EETALAAFLSYCPGMGVALRMYPLKVTWWFCAFPYSLLIFIYDEVRKLILRRYPGGWVEK 1017

human2 EETALAAFLSYCPGMGVALRMYPLKVTWWFCAFPYSLLIFIYDEVRKLILRRYPGGWVEK 1016

mouse2 EETALAAFLSYCPGMGVALRMYPLKVTWWFCAFPYSLLIFIYDEVRKLILRRYPGGWVEK 1016

rabbit2 EETALAAFLSYCPGMGVALRMYPLKVTWWFCAFPYSLLIFIYDEVRKLILRRYPGGWVEK 1016

pig2 EETALAAFLSYCPGMGVALRMYPLKVTWWFCAFPYSLLIFIYDEVRKLILRRYPGGWVEK 1016

cow2 EETALAAFLSYCPGMGVALRMYPLKVTWWFCAFPYSLLIFIYDEVRKLILRRYPGGWVEK 1016

elephant2 EETALAAFLSYCPGMGVALRMYPLKVTWWFCAFPYSLLIFLYDEVRKLILRRYPGGWVEK 1015

pig1 EETALAAFLSYCPGMGVALRMYPLKPTWWFCAFPYSLLIFVYDEVRKLIIRRRPGGWVEK 1017

human1 EETALAAFLSYCPGMGVALRMYPLKPTWWFCAFPYSLLIFVYDEVRKLIIRRRPGGWVEK 1019

dog1 EETALAAFLSYCPGMGVALRMYPLKPTWWFCAFPYSLLIFVYDEVRKLIIRRRPGGWVEK 1017

cow1 EETALAAFLSYCPGMGVALRMYPLKPTWWFCAFPYSLLIFVYDEVRKLIIRRRPGGWVEK 1017

sheep1 EETALAAFLSYCPGMGVALRMYPLKPTWWFCAFPYSLLIFVYDEVRKLIIRRRPGGWVEK 1017

mouse1 EETALAAFLSYCPGMGAALRMYPLKPTWWFCAFPYSLLIFVYDEVRKLIIRRRPGGWVEK 1019

rabbit1 EETALAAFLSYCPGMGVALRMYPLKPTWWFCAFPYSLLIFVYDEIRKLIIRRRPGGWVEK 1019

Elephant1 EETALAAFLSYCPGMGVALRMYPLKPTWWFCAFPYSLLIFVYDEVRKLIIRRRPGGWVEK 1017

horse1 EETALAAFLSYCPGMGVALRMYPLKPTWWFCAFPYSLLIFVYDEVRKLIIRRRPGGWVEK 1017

****************.******** **************:***:****:** *******

111111111111111111111111151111111111111131111111131151111111

dog2 ETYY 1020

horse2 ETYY 1021

human2 ETYY 1020

mouse2 ETYY 1020

rabbit2 ETYY 1020

pig2 ETYY 1020

cow2 ETYY 1020

elephant2 ETYY 1019

pig1 ETYY 1021

human1 ETYY 1023

dog1 ETYY 1021

cow1 ETYY 1021

sheep1 ETYY 1021

mouse1 ETYY 1023

rabbit1 ETYY 1023

Elephant1 ETYY 1021

horse1 ETYY 1021

****

1111
